# Astrocyte Ca^2+^-evoked ATP release regulates myelinated axon excitability and conduction speed*

**DOI:** 10.1126/science.abh2858

**Published:** 2021-10-15

**Authors:** Jonathan Lezmy, Lorena Arancibia-Carcamo, Tania Quintela-Lopez, Diane L. Sherman, Peter J. Brophy, David Attwell

**Affiliations:** 1 Department of Neuroscience, Physiology and Pharmacology, University College London, London, WC1E 6BT, UK; 2 Dementia Research Institute, Francis Crick Institute 1 Midland Rd, London, NW1 1AT, UK; 3 Centre for Discovery Brain Sciences, University of Edinburgh, Chancellor’s Building, Edinburgh, EH16 4SB

## Abstract

**Introduction:**

Astrocytes support neuronal function throughout the central nervous system. In the grey matter they regulate synapse number during development, remove synaptically-released neurotransmitters to terminate their action and prevent excitotoxicity, control the extracellular potassium concentration to prevent hyperexcitability, regulate blood flow to ensure an adequate energy supply, provide lactate to neurons for energy, and respond to rises of intracellular calcium concentration ([Ca^2+^]_i_) by releasing ATP and other gliotransmitters that act on neuronal receptors to modulate information processing. However, their role is unclear in the white matter, which transmits information rapidly between grey matter areas using axons wrapped with capacitance-reducing myelin, although they have been suggested to regulate myelination during development and during normal function.

**Rationale:**

Recently it has been suggested that learning and memory may reflect, not only changes of synaptic function in the grey matter, but also changes of white matter function. In particular, neural circuit function might be regulated by changes in the conduction speed of myelinated axons that result in an altered arrival time of action potentials at a distant neuron. These speed changes might be brought about by alterations of the properties of the passively conducting myelinated internodes, or of the intervening excitable nodes of Ranvier where the action potential is generated. We applied immunohistochemistry to assess how astrocytes interact with myelinated axons, used neuronal stimulation and light evoked calcium uncaging in astrocytes to evoke Ca^2+^-dependent release of gliotransmitters, and employed electrophysiology and pharmacology to characterise how astrocyte-released substances might affect the axon initial segment (AIS) and nodes of Ranvier of myelinated neurons. Measurements of conduction velocity and computer modelling allowed us to interpret the results.

**Results:**

Astrocytes closely approach the axons of myelinated neurons in layer V of the cerebral cortex. Uncaging Ca^2+^ within astrocytes, or stimulating spike trains in neurons, evoked a rise of astrocyte [Ca^2+^]_i_ that triggered the release of ATP-containing vesicles from these cells. This evoked an inward current in the AIS and nodes of Ranvier of the pyramidal neurons. Pharmacology showed that this was mediated by the activation of G_s_-linked adenosine A_2a_ receptors, implying that the released ATP was converted to adenosine by extracellular enzymes. The A_2a_ receptors raise the intracellular concentration of cyclic AMP, which activates HCN channels mediating the inward hyperpolarization-activated current I_h_, and thus depolarizes the cell. In the AIS the activation of A_2a_ receptors alters excitability and hence action potential generation, while in the nodes of Ranvier it decreases the conduction speed of the action potential along the axon.

**Conclusion:**

As in the grey matter, astrocyte [Ca^2+^]_i_ regulates the release of ATP into the extracellular space in the white matter. After conversion to adenosine, this regulates the excitability and conduction speed of myelinated axons. The changes in excitability at the AIS will lead to changes in the relationship between the synaptic input and action potential output of the cell. The altered conduction speed of the myelinated axon may change neural circuit function by changing the action potential arrival time at the cell’s output synapses, thus altering the integration of signals in postsynaptic neurons. Variations in astrocyte-derived adenosine level can occur between wake and sleep states, and the extracellular adenosine concentration rises during energy deprivation conditions. These changes of adenosine level could thus control white matter information flow and neural circuit function.

In the brain’s grey matter, astrocytes regulate synapse properties, but their role is unclear for the white matter where myelinated axons rapidly transmit information between grey matter areas. We found that, in rodents, neuronal activity raised [Ca^2+^]_i_ in astrocyte processes located near action potential generating sites in the axon initial segment (AIS) and nodes of Ranvier of myelinated axons. This released ATP which was converted extracellularly to adenosine and thus, via A_2a_ receptors, activated HCN2-containing cation channels that regulate two aspects of myelinated axon function: excitability of the AIS and speed of action potential propagation. Variations in astrocyte-derived adenosine level, between wake and sleep states or during energy deprivation, could thus control white matter information flow and neural circuit function.

**Figure:**
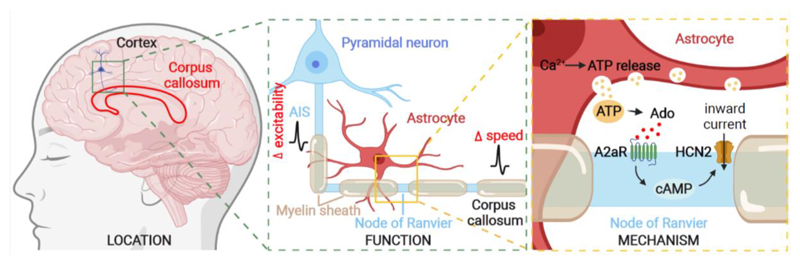
Astrocytes regulate myelinated axon excitability and conduction speed. For cortical neurons with myelinated axons crossing the corpus callosum (left), astrocytes regulate axon initial segment (AIS) excitability and axonal conduction speed (middle). Increases of astrocyte [Ca^2+^]_i_ release ATP which, after conversion to adenosine (Ado) extracellularly, activates A_2a_ receptors that raise the intracellular cyclic AMP concentration, and thus generate an inward current via HCN2 channels in the AIS and nodes of Ranvier (right). Created with BioRender.com.

Astrocytes support neuronal function throughout the central nervous system. In the grey matter they regulate synapse number during development, remove synaptically-released neurotransmitters, control the extracellular [K^+^] ([K^+^]_o_), regulate blood flow and provide lactate to neurons for energy (1). However, their role is unclear in the white matter, which transmits information rapidly between grey matter areas using myelinated axons. Wrapping of these axons with myelin by oligodendrocytes reduces the axon capacitance and thus confers a high conduction speed for the action potential which, once generated at the axon initial segment (2), is maintained as it propagates by sodium influx at the nodes of Ranvier occurring between the myelinated internodes. Because oligodendrocytes isolate almost all of the axon from the extracellular space, they are thought to be the main mediators of [K^+^]_o_ control and energy provision to axons (3, 4), making the role of astrocytes uncertain, despite astrocyte processes occurring close to nodes of Ranvier (5).

Labeling for GFAP revealed astrocytes throughout the grey matter and white matter (Fig. 1A). On patch-clamping layer V cortical pyramidal cells or oligodendrocytes we observed that astrocyte processes are aligned and intimately associated with the myelinated axon and its internodal sheaths (Fig. 1B, C, Fig. S1A-F). We used double whole-cell patch-clamping to load an astrocyte with a Ca^2+^-sensing dye (Fluo-4), and to depolarize a pyramidal cell to stimulate action potentials. When the pyramidal cell was driven to fire briefly (30 Hz for 1 sec) the astrocyte intracellular calcium concentration ([Ca^2+^]_i_) rose more in processes near the neuronal dendrites (within 12.7±2.5 sec from start of stimulus to peak ΔF/F; mean ΔF/F=0.20±0.03, n=5 neuron-astrocyte pairs) than in processes near the axon (ΔF/F=0.03±0.02, p=0.023; Fig. 1E). However, if prolonged spiking was evoked (10 sec), [Ca^2+^]_i_ rose in both locations (Fig. 1D, E; movie S2; ΔF/F= 0.19±0.08 and 0.17±0.05 respectively, p=0.63, n=3). Uncaging Ca^2+^ in the astrocyte soma also raised astrocyte [Ca^2+^]_i_ (Fig. 1F, ΔF/F= 1.06±0.51 near dendrite, ΔF/F=1.05±0.53 near axon, p=0.86, n=6), and neuronal activity evoked a further [Ca^2+^]i rise superimposed on this (ΔF/F=0.39±0.09 near dendrite, ΔF/F=0.01±0.06 near axon, p=0.018).

In the grey matter, astrocytes can modulate neuronal function by releasing ATP (6). We imaged putative vesicles containing ATP in astrocytes around myelinated axons of layer V pyramidal neurons using quinacrine (7). Uncaging Ca^2+^ in the astrocyte soma evoked a Ca^2+^ wave that propagated into processes along the axon (Movie S2), and triggered the loss of 43% of quinacrine-labeled vesicles (Fig. 1G-I; p=0.0053); a loss which was not seen when the 2-photon uncaging excitation was insufficient (see Methods, Fig. S2) to evoke a detectable rise of [Ca^2+^]_i_ nor when assessing ATP vesicle number in areas 5 μm away (Fig. 1J, K). The resulting ATP release into the extracellular solution was detected using luciferin-luciferase (see Methods). Puffing 1 mM ATP, but not aCSF, from a pipette into the sensing solution evoked a large increase of luciferin luminescence (Fig. 2A), while uncaging Ca^2+^ in an astrocyte soma evoked a similar response that was not seen when the uncaging illumination was too weak to evoke a detectable [Ca^2+^]i rise (Fig. 2B, C).

On release, ATP is rapidly hydrolysed to adenosine by ecto-ATPases located on microglia and astrocytes (8). Microglia and astrocytes associate with both the AIS and nodes of Ranvier (9, 10). Adenosine receptors have previously been reported at synapses. We used immunohistochemistry to identify adenosine receptors on layer V neuron myelinated axons. Neither A_1_ nor A_2b_ receptors were detected (Fig. S1G, H), but A_2a_ receptors (A_2a_Rs) were found at 92% of AISs (Fig. 2D, E, H) and at 85% of nodes of Ranvier (Fig. 2F-H). Overview images of the cortex (Fig. S3D) showed A_2a_R-expressing AISs leading towards the corpus callosum, implying that these were excitatory neurons. A_2a_Rs were detected in less nodes of the cerebellar white matter which contains excitatory mossy and climbing fibre axons as well as inhibitory Purkinje cell axons (70%, Fig. S3F), and were absent from AISs of cerebellar Purkinje cells (Fig. S3E), implying a neuron-type specific expression of A_2a_R in myelinated axons. A_2a_Rs raise cyclic AMP level, which can affect cell excitability by promoting the opening of hyperpolarization-activated HCN type channels (11, 12), which are present in axons (13–15). We detected HCN2 channel subunits at 51% of AISs and 64% of nodes of Ranvier (Fig. 2I-M). HCN1 subunits were observed in pyramidal cell somata and in nodes of Ranvier (Fig. S1G-I). Adenosine receptors and HCN channels have not previously been reported at the node of Ranvier.

To examine the effect of activation of these A_2a_Rs we patch-clamped layer V pyramidal neurons and applied adenosine (100 μM from a puffer pipette), or another agonist for A_2a_Rs (CGS 21680, 0.5 μM), to the AIS (Fig. 3A). Activating A_2a_Rs in this way depolarized the cell by 6-7 mV (Fig. 3A, B, E; aCSF had no effect). The action potential response to small injected currents was increased in frequency (Fig. 3C, F, G), but at high injected currents was reduced presumably as a result of increased Na^+^ channel inactivation (Fig. 3D, F, H). Hyperpolarizing the cell evoked a time-dependent inward current increase (Fig. 3I), which was mediated by I_h_ (HCN) channels, because it was blocked by the I_h_ blocker ZD7288 (Fig. S4). Applying CGS 21680 increased the amplitude of I_h_ tail currents, reflecting an increase in magnitude of the fully activated conductance of 51% in 6 cells (p=0.029), and shifted the 50% point of the activation curve derived from these currents in the depolarizing direction by +8.2 mV (p=0.045) so that more I_h_ is activated in the physiological range (Fig. 3I-K). This shift was mimicked by including 50 μM cAMP in the patch pipette (shifted by +8.6 mV in 6 cells, p=0.026) and in these cells CGS 21680 evoked no further depolarizing shift (-1.77 mV in 6 cells, p=0.087, Fig. 3J-L).

To assess the function of A_2a_Rs at the nodes of Ranvier, we patch-clamped layer V pyramidal cells. Dye filling revealed an enlarged bleb at the end of the axon where it was cut during the brain slicing process. This allowed patch-clamping of the bleb (see Materials and Methods), which was ~3 (mean 3.2±0.4 in 5 cells) nodes away from the soma (Fig. 4A). In Thy1-Caspr-GFP mice, the nodes of layer V pyramidal neurons could be seen during the experiment, from paranodal GFP fluorescence and because axon branches often occur at nodes, while post-recording immunohistochemistry revealed A_2a_Rs at the AIS and nodes (Fig. 4A). Puffing CGS 21680 onto a node (Fig. 4A) did not significantly depolarize the pyramidal cell soma (0.99±0.46 mV in 5 cells, p=0.1), implying that the puffed drug did not reach the soma or AIS. Injecting current (500 pA for 5 msec, repeated at 2 Hz) into the soma evoked an action potential. This evoked a delayed action potential in the axonal bleb, which became more delayed when CGS 21680 was puffed at an intervening node (Fig. 4B; >100 responses were averaged). We used plots of d(signal)/dt versus signal (Fig. 4C) to estimate when the action potentials in each location showed an accelerating onset phase (soma depolarization >1 mV/msec and a bleb inward current increase more negative than -0.5 pA/msec, shown as dashed lines in Fig. 4B). The latency of the response at the bleb increased from 108±31 μsec in control conditions to 320±89 μsec when CGS 21680 was puffed (p=0.03, paired *t*-test, n=5 cells, Fig. 4D) and the spike width recorded at the bleb increased by 0.28 msec from 0.30±0.10 msec to 0.58±0.14 msec (p=0.015, paired *t*-test, n=5).

To convert this latency increase to a change of conduction speed we need to assume where in the AIS the action potential is initiated, and how fast it propagates backwards to the soma compared to forwards to the bleb (see Materials and Methods). If the spike starts at the middle of the AIS and the forward speed is twice the backward speed (16), then CGS 21680 reduced the forward speed from 1.21±0.23 m/sec to 0.43±0.08 m/sec (64% reduction, filled circles in Fig. 4E). Alternatively, if the spike starts at the end of the AIS and the forward speed is three times faster than the backward speed (16) then the speed is reduced from 0.68±0.16 m/sec to 0.31±0.13 m/sec (54% reduction, open circles in Fig. 4E). Thus, for a range of assumptions, activation of nodal A_2a_Rs produces a very significant reduction of conduction velocity (p=0.0017 comparing the ratio of the conduction velocities).

To explore how A_2a_Rs and I_h_ affect excitability and conduction velocity, we employed a MATLAB model of myelinated axons in the corpus callosum (17, 18), which was adapted to mimic either (i) a soma attached to a truncated axon with 3 internodes and a terminal bleb (Fig. 5A), or (ii) an infinitely long axon (see Materials and Methods). The AIS and nodes of Ranvier contained voltage-gated Na^+^ and K^+^ channels to generate action potentials, while the soma and bleb lacked these channels. We modeled the I_h_ current in the absence of A_2a_R activation as being present in the soma and proximal AIS only and unaffected by the puff application of A_2a_R activating drugs at the distal AIS (and the [cAMP] rise that they produce), consistent with the presence in the somatodendritic compartment (Fig. S1G) of I_h_ channels composed of HCN1 subunits (19), which are relatively insensitive to cAMP (20). The increase in I_h_ evoked by A_2a_R activation in the AIS was modeled as the addition of an extra current mediated by cAMP-activated HCN2 channels located in the distal AIS. The maximum conductance of this extra current was set to increase the total maximum I_h_ conductance (measured at the soma) by 51% as observed (Fig. 3I), and the midpoint of the activation curve was set at -80 mV to reproduce the positive shift for the total I_h_ apparent activation curve in Fig. 3J. Adenosine-activated I_h_ was also added to the nodes of Ranvier at a density 1.41-fold higher than in the distal AIS, as measured immunohistochemically (see Materials and Methods).

Adding the adenosine-activated conductance to the distal AIS in the model depolarized the soma by 5.9 mV, as found experimentally (Figs. 3E, 5B). It also increased the firing evoked by a small injected current (Fig. 5C, D), while decreasing that evoked by a large current (Fig. 5C, D), as seen experimentally (Fig. 3C-H). However, the simulated depression of action potential amplitude at large currents was larger than that observed experimentally. These changes were largely a result of the I_h_ added to the AIS, because adding it solely to the nodes had only a minor effect on the resting potential at the soma (Fig. 5B). Adding I_h_ to the nodes of Ranvier decreased the conduction speed (Fig. 5E) as observed when puffing CGS 21680 onto the nodes (Fig. 4E). The predicted reduction was smaller than that observed experimentally, presumably due to the mild depolarization generated at the nodes (+3.8 mV). Using the infinite cable model, adding the adenosine-activated I_h_ depolarized the nodes by +11.4 mV, reduced the predicted conduction speed by 48%, from 2.23 to 1.17 m/s (Fig. 5F) and increased the axonal spike width by 0.22 ms (mainly by increasing Na^+^ channel inactivation at the resting potential because this was mimicked by decreasing the peak Na^+^ conductance ~2-fold), similar to the 0.28 msec reported experimentally above.

Because astrocyte [Ca^2+^]_i_ rises evoked the release of ATP (Fig. 1G-K; Fig. 2A-C), which is expected to be converted to adenosine extracellularly, and to act on A_2a_Rs at the AIS and nodes of Ranvier, we tested whether uncaging Ca^2+^ in astrocytes modulates the excitability and conduction speed of cortical layer V pyramidal cell myelinated axons. Ca^2+^ was uncaged in an astrocyte with processes running near the AIS of a pyramidal cell (Fig. 6A) (movie S3). Within 70 sec of uncaging the Ca^2+^, the resting potential of the neuron was depolarized (Fig. 6B) and the action potential response of the neuron was changed (Fig. 6C), exhibiting a higher firing rate to low injected currents (p = 0.0175 when comparing firing rates normalised to the maximum rate evoked at high injected current), and a lower firing rate to high injected currents (p=0.0136). These changes, which are similar to those seen when activating AIS A_2a_Rs (Fig. 3B-H), were not mediated by glutamate release from astrocytes (Fig. S5), were blocked by superfusion of the A_2a_R blocker ZM 241385 (100 nM) or by intracellular dialysis of the neuron with the I_h_ blocker ZD7288 (20 μM), and were not seen if the uncaging illumination failed to evoke a [Ca^2+^]_i_ rise (Fig. 6B-F).

Uncaging Ca^2+^ in astrocytes that were shown, by *post hoc* immunostaining, to have processes close to nodes of Ranvier (Fig. 6G, H), evoked a decrease of axonal conduction speed (Fig. 6I) in experiments which monitored action potential propagation from the soma to a bleb ~4 (mean 3.8±0.5 in 5 cells) nodes along the axon. These data are similar to those obtained by puffing an A_2a_R agonist onto the nodes (Fig. 4) and, as for those experiments, the decrease of conduction speed calculated is dependent on the exact site of action potential initiation within the AIS and its forward and backward propagation speeds (Fig. 6I). Introducing ZD7288 (20 μM) into the targeted axons to block I_h_ (HCN) channels prevented the speed reduction evoked by astrocyte Ca^2+^ activity (Fig. S6A). In unmyelinated axons and some other neuronal types, axonal HCN channels can speed, rather than slow, the action potential (13, 14) (see Fig. S6 legend). Inducing robust neuronal activity (30 Hz for 1 min) to raise astrocyte [Ca^2+^]_i_ (Fig. 1E) also induced a decrease of axonal conduction speed (p=0.014), which was abolished by superfusing the A_2a_R blocker ZM 241385 (100 nM) (Fig. S6B).

These data reveal a novel modulation of neuronal circuit function by glial cells. Astrocyte modulation of neuronal synaptic function in the grey matter has been accepted as a major determinant of neuronal function (6, 21, 22), but the role of white matter astrocytes is less clear. Our data, summarized in Fig. S7, demonstrate that astrocytes modulate the excitability of the axon initial segment of excitatory neurons, and regulate the conduction speed of myelinated axons. This regulation is a result of astrocytes raising the concentration of adenosine near the AIS or nodes of Ranvier (of a single axon or possibly several axons at once), which can increase or decrease AIS excitability, and which decreases axon conduction speed. Adenosine release can occur when axonal action potential firing raises [Ca^2+^]_i_ in adjacent astrocytes (23), which triggers the release from vesicles of ATP (Figs. 1 & 2), that is subsequently converted to adenosine by ecto-ATPases expressed by microglia, astrocytes and oligodendrocyte lineage cells (8). Neuronal activity in cortical layer V grey matter readily raised [Ca^2+^]_i_ in astrocyte processes near neuronal dendrites, presumably as a result of classical neurotransmitters or other signaling molecules acting on astrocytes, but more intense neuronal activity (or conceivably a large number of neurons firing at a low rate) was needed to raise it in the processes near axons (Fig. 1E-F). Thus, the overall level of neuronal activity may modulate the conduction speed of white matter axons.

Changes of ATP level in astrocytes or neurons may also alter the extracellular adenosine concentration, as a result of intracellular interconversion of ATP and adenosine, and export or import of adenosine across the cell membrane by the equilibrative nucleoside transporter. Across a wide area of neocortex, intracellular ATP level decreases during neuronal activity, on passing from the awake state to non-REM sleep, and on passing from non-REM sleep to REM sleep (24). In the basal forebrain accumulation of extracellular adenosine when awake has been proposed to generate pressure to sleep (25), and this adenosine is likely to be generated from ATP released by astrocytes (26, 27). Although the arousal-modulating effects of adenosine have often been attributed to it acting on presynaptic A_1_ receptors to suppress glutamate release at excitatory synapses, genetic evidence suggests that in fact these effects are generated by A_2a_Rs (28). Thus, the effects of astrocyte-derived adenosine on AIS excitability and myelinated axon conduction speed that we have characterised may be crucial contributors to mediating changes between wake and sleep states.

GABA, dopamine and serotonin receptors regulate neuronal excitability at the AIS (15,29,30). Our data demonstrate that the node of Ranvier can have its electrical function rapidly altered by substances released from surrounding cells. We have shown this for astrocyte-released ATP/adenosine, but oligodendrocyte precursor cells and microglia could theoretically exert similar effects, either by releasing ATP/adenosine directly onto spike generation sites or by responding to astrocytic ATP release by releasing other gliotransmitters onto the axons. Neural circuits depend crucially on action potential arrival time for their function (31), and cognition depends on oscillations of neural firing probability that in turn depend on propagation time in myelinated neurons (32–34). Thus, the decrease of the conduction speed of myelinated axons that is evoked by adenosine may change information processing and cognition, when the adenosine level rises either during a prolonged awake period or in response to complex motor behaviour and pathological conditions. Indeed, behavioral effects linked to exploratory activity, aggression and anxiety (35, 36), and pathological effects in epilepsy, Parkinson’s disease, Alzheimer’s disease, depression and autism have all been linked to impairments of adenosine signalling (37, 38). Our results make the testable prediction that, when adenosine levels rise during prolonged wakefulness, or in complex motor behaviour and pathological conditions, the conduction speed of myelinated axons should decrease, resulting in delays to the action potential arrival time at downstream synapses and possible impairments of coincidence detection or oscillatory firing generation.

## Materials and Methods

### Animals

Sprague-Dawley rats or transgenic mice aged P28-32, housed on a 12 h/12 h light/ dark cycle were used in all experiments. At this age, myelination is advanced but cells are still suitable for stable patch-clamp recordings. Animals were killed to make brain slices 4 h after the room light was turned on. Each experiment was performed on brain slices from at least three animals and at least one of each sex. Expression of A_2a_Rs and HCN channels in sites of spike generation appeared the same in mouse and rat and in males and females. Animal procedures were carried out in accordance with the guidelines of the UK Animals (Scientific Procedures) Act 1986 and subsequent amendments (under Project Licence 70/8976). Thy1-Caspr-GFP mice were generated as previously described (39).

### Acute brain slice preparation

300 μm thick coronal cortical slices were prepared on a vibratome in ice-cold solution containing (in mM): 93 N-methyl-D-glucamine (NMDG) chloride, 2.5 KCl, 30 NaHCO_3_, 10 MgCl_2_, 1.2 NaH_2_PO_4_, 25 glucose, 0.5 CaCl_2_, 20 HEPES, 5 sodium ascorbate, 3 sodium pyruvate and 1 kynurenic acid. The slices were incubated at 37°C in this solution for 20 min, then transferred to a similar solution with (mM) 93 NaCl, 1 MgCl_2_ and 2 CaCl_2_ instead of the NMDG chloride, MgCl_2_ and CaCl_2_, and incubated at room temperature until use. Experiments were performed in artificial cerebrospinal fluid (aCSF) containing (in mM): 125 NaCl, 3 KCl, 26 NaHCO_3_, 2 MgCl_2_, 2 CaCl_2_, 1.25 NaH_2_PO_4_ and 10 glucose, heated to 37°C. All the solutions were gassed with 95% O_2_ and 5% CO_2_. In some experiments, 50 μM ZD7288 (Tocris), 100 nM ZM 241385 (Tocris), 20 μM D-AP5 (Tocris), 10 μM DNQX (Tocris), 50 μM MSPG (Tocris) and 1 μM NPS 2390 (Tocris) were added to the aCSF.

### Patch-clamping of neuronal somata

Experiments were performed with an Olympus BX51WI microscope, under an Olympus LUMPlanFI x40 lens. Layer V pyramidal cells were identified by their location and morphology. Microelectrodes with resistances of 5-6 MΩ were pulled from borosilicate glass capillaries (Harvard Apparatus) and filled with an intracellular solution containing (in mM): 145 K-gluconate, 2 MgCl_2_, 0.5 H_2_-EGTA, 2 MgATP, 0.2 Na_2_GTP, and 10 HEPES, pH adjusted to 7.2 with KOH. Somata were patch-clamped in whole-cell configuration, and the signals were amplified using a Multiclamp 700B (Molecular Devices), filtered at 4 kHz and digitised at 50 kHz. The series resistance was less than 20 MΩ, was compensated by at least 70%, and did not vary by more than 10% during experiments. The mean input resistance in whole-cell mode, after compensation for series resistance, was 130.6±25.9 MΩ in control conditions and 84.8±23.8 MΩ during CGS 21680 application to the AIS (n=6, p=0.049). Recordings were acquired with Clampex and analysed with Clampfit software (Molecular Devices). In current-clamp mode, bridge balance and pipette capacitance were corrected. To generate graphs of firing rate versus injected current, current steps in increments of 100 pA were injected for 1 sec. All membrane potentials were corrected for a pipette liquid junction potential of -16 mV. To analyse currents activated by hyperpolarization, we analysed the tail currents at -136 mV obtained following 1 sec pre-test pulses in 10 mV increments from -56 mV to -146 mV. The tail currents analyzed were primarily generated by HCN channels (I_h_ current) because they were significantly diminished by adding 50 μM ZD7288 (Tocris) (Fig. S4). Tail currents were fitted with single exponential curves that were extrapolated back to the end of the pre-test pulses. The resulting tail amplitude values were fit to a function:

(1) $$I=I_{max}+\left(I_{min}\text{-}I_{max}\right)/\left(1+exp\left(k\left(V\text{-}V_{1/2}\right)\right)\right)$$

where *I_max_* is the predicted tail amplitude at -136 mV for a large depolarizing voltage step, *I_min_* is the tail amplitude for a large hyperpolarizing step, *V_1/2_* is the voltage mid-point of the activation curve and *k* is a factor defining the slope of the activation curve. The conductance conferred by the maximally activated current is given by:

(2) $$G_{max}=\left(I_{max}\text{-}I_{min}\right)/\left(V_{rev}\text{-}\left(\text{-}136\;mV\right)\right)$$

where the reversal potential of I_h_ was taken as -23 mV (13). In some experiments, 50 μM cAMP (Sigma) or 20 μM ZD7288 (Tocris) were added to the intracellular solution. ZD7288 was previously shown to block I_h_ when applied intracellularly (15).

### Patch-clamp recording from axonal blebs

Experiments were performed using a Zeiss LSM780 two-photon/confocal microscope, with a W Plan-Apochromat 20x objective. Blebs are patchable structures formed at the cut end of axons, at the surface of slices (40). Alexa 594 (ThermoFisher) was allowed time to diffuse from the soma to the bleb. Action potentials were recorded as a current in voltage-clamp mode. To avoid phototoxicity, exposures to excitation lights were briefly used only to trace the axon and monitor the puffing area (see below). High-resolution images of whole axons were acquired after completion of the electrophysiology experiments. Exposure to the excitation lasers did not significantly affect the waveform of action potentials. In Fig. 6D-E, where the effects of adenosine receptors or HCN channels were blocked, there was no change in action potential width measured at the soma. For the first spike evoked by a 500pA step, the full width at half-maximum with ZM 241385 was 1.15±0.13 msec before and 1.18±0.14 msec after Ca^2+^ uncaging (n=4; not significantly different, p=0.12), and with ZD7288 was 1.25±0.17 msec before and 1.21±0.13 msec after Ca^2+^ uncaging (n=4; not significantly different, p=0.44). Similarly, there was no difference in the action potential width measured at the bleb with ZD7288 inside the neurons (Fig. S6A): the full width at half-maximum (the maximum being the amplitude of the inward current peak) was 0.53±0.17 msec before and 0.53±0.10 msec after Ca^2+^ uncaging (n=5; not significantly different, p=0.99). Microelectrodes with resistances of 8-9 MΩ were filled with aCSF and 100 μM Alexa 488 (ThermoFisher). Blebs were patch-clamped in cell-attached configuration, the signals were filtered at 2 kHz and digitised at 50 kHz. Action potentials at the soma were evoked and recorded, and action currents at the axon bleb were recorded simultaneously. In order to accurately determine the delay between the somatic and axonal signals, at least 100 somatic spikes were evoked, first derivatives of the somatic and axonal signals were generated and they were time-shifted to align the peaks of the somatic spikes. The signals were then averaged and the soma-axon delay was measured as the difference between the onset of the axonal spike (time point recorded before a current drop of >0.5 pA/ms) and the somatic spike (time point recorded before an increase of >1 mV/ms).

### Local application of drugs to axons

500 nM CGS 21680 (Cayman) or 100 μM adenosine (Sigma) were puff-applied onto the AIS or nodes of Ranvier. The drugs were added to aCSF with 100 μM Alexa 594 to monitor the spread of the drugs. 2-3 MΩ pipettes were filled with this solution and placed 10 μm away from the targeted axon. Positive pressure was applied either with a micro-injector (PMI-100, Dagan) for 20 ms at 10 psi or controlled manually with a syringe. In both cases, positive pressure was calibrated and live monitored to eject drug over a radius of 20 μm or less (from the tip of the pipette set at saturating intensity to the edge of detectable fluorescence). The flow of the perfusion was set to wash out the puffed drug in the direction away from the targeted neuron. The AIS was detected by adding 100 μM Alexa 594 into the intracellular solution and its diffusion into the axon was live monitored. The pipette puffing onto the AIS was placed near the distal AIS 25-30 μm away from the soma. The pipette puffing onto the nodes was placed near an axonal branch (detected from the Alexa 594 fill) or green GFP signal detected along the axon of a Thy1-Caspr-GFP mouse. Caspr was *post hoc* immunolabeled to confirm the presence of nodes at the puffing sites.

### Patch-clamping, calcium uncaging and imaging of astrocytes

Astrocytes were detected by their morphology and with the selective marker Sulforhodamine 101 (SR 101). Before the experiments, the slices were incubated with 1 μM SR101 (Tocris) added to the slicing solution for 20 minutes at 37 °C. We confirmed the presence of astrocytes by *post hoc* fixing slices and immunolabeling for GFAP. Microelectrodes with resistances of 7-8 MΩ were filled with a K-methylsulfate intracellular solution containing (in mM): 100 KMeSO_4_, 50 KCl, 2 MgCl_2_, 4 MgATP, 0.3 Na_2_GTP, and 10 HEPES. 100 μM Fluo-4 (ThermoFisher) was added to image calcium changes and 5 mM NP-EGTA (ThermoFisher) was added to allow uncaging of calcium in single astrocytes. Images were acquired at 2 Hz with a confocal argon laser and NP-EGTA photolysis was obtained by applying 2-photon excitation at 720 nm to the soma (10 iterations, 2.55 μs pixel dwell time). Laser intensity was set at 10 mW and, if needed, was increased gradually until a calcium concentration rise was evoked (Fig. S2). Intensities just below those needed to evoke a detectable [Ca^2+^]_i_ rise were used as control experiments to check that 2-photon illumination alone was not responsible for the release of ATP and adenosine generation observed. In the experiments examining calcium activity at astrocyte processes, images were acquired at the plane of the axon or of a dendrite contacting astrocyte processes. Regions of interest (ROIs) were selected and [Ca^2+^]_i_ changes were measured as fractional changes in the fluorescence signal (ΔF/F) after background subtraction. At the start of the experiments, the baseline astrocyte Ca^2+^ activity was recorded for 10 sec and, over this brief period, no spontaneous Ca^2+^ transients were observed.

### Immunohistochemistry

Brains from Thy1-Caspr-GFP mice were perfusion-fixed in 4% paraformaldehyde (PFA) in 0.01 M phosphate buffer saline (PBS) and fixed tissue was then cut into 70 μm-thick slices. Alternatively, 300 μm-thick acute slices from mouse and rat brains were immersion-fixed in a solution containing 4% PFA, 4% sucrose, and 0.1M PBS. The slices were permeabilized with 0.2% Triton X-100 (Sigma) in a blocking solution (10% goat serum in 0.01 M PBS) for 1 h. The slices were then incubated overnight at 4 °C with the following primary antibodies, as required: rabbit anti-A_2a_R (Abcam, ab3461, 1:100), mouse anti-A_2a_R (Millipore, 05-717 which has been validated as giving no labelling in A_2a_R KO tissue (41), 1:200), rabbit anti-A_1_R (Abcam, ab82477, 1:100), rabbit anti-A_2b_R (Cohesion, CPA3755, 1:100), mouse anti-Ankyrin G (Neuromab, N106/36, 1:500), mouse anti-panNav (Sigma, K58/35, 1:100), mouse anti-Caspr (Neuromab, K65/35, 1:100), chicken anti-GFP (Millipore, AB16901, 1:1000), rabbit anti-GFAP (Millipore, AB5804, 1:500), rabbit anti-HCN1 (Alomone, APC 056, 1:100) and rabbit anti-HCN2 (Alomone, APC 030, 1:100). The slices were then incubated for 2 hours at room temperature with the following secondary antibodies, as required (ThermoFisher, 1:500): anti-chicken or anti-mouse Alexa Fluor 488, anti-rabbit or anti-mouse Alexa Fluor 546 and anti-rabbit Alexa Fluor 647. The slices were incubated with DAPI nuclear stain (1:50,000 in PBS from a 5 mg/ml stock concentration) for 10 min and were mounted in Dako Fluorescent Mounting Medium. Slices were washed with 0.01M PBS three times for 10 minutes between each step. Negative control experiments were also carried out to check for any labeling caused by unspecific binding of the secondary antibodies: for that purpose we followed the same protocol but omitting the incubation with the primary antibodies. Slices were imaged using a Zeiss LSM700 confocal microscope with a Zeiss Plan-Apochromat 63x oil immersion lens, and images were acquired with ZEN Microscope Software (Zeiss). Z-stacks of 1 μm-interval were imaged and their maximum intensity was projected using ImageJ (FIJI). A line was drawn along the axons to plot intensity profiles. To plot node of Ranvier profiles, the width of the line was adjusted to fit the width of the Caspr-labeled paranodes. To analyse GFAP staining running parallel to axons, the width of the line was adjusted to be 5 μm and centred on the axon.

### Quantification of putative ATP-containing vesicles

Before the experiments, the slices were incubated with 20 μM quinacrine dihydrochloride (Sigma) added to the slicing solution for 25 minutes at 37 °C. Quinacrine is a green selective marker for ATP-containing vesicles (7) or lysosomes (42). Calcium was uncaged and imaged from single astrocytes as described above, using the red Ca^2+^ indicator Rhod-2 (50 μM, ThermoFisher) instead of Fluo-4. Images of astrocyte processes with ATP vesicles were acquired before and after Ca^2+^ uncaging with 2-photon excitation at 720 nm at the soma. Adjacent areas were also imaged: we selected ROIs located a similar distance away from the astrocyte soma but without any detectable dye-filled astrocyte processes within a 5 μm perimeter minimum. With ImageJ, a constant threshold was applied to the images and particle analysis was used to quantify the number of ATP vesicles. The mean Feret’s diameter of ATP vesicles was 0.56±0.03 μm (n=2227), similar to previous reports (43).

### Detection of extracellular ATP

Changes in extracellular ATP level were detected with a luciferin/luciferase-based chemiluminescence assay emitting green light. Perfusion of brain slices was halted and 50 μl aliquots containing 12 mg/ml luciferin (Sigma) and 5 mg/ml luciferase (Sigma) were added to a 1 ml bath chamber filled with aCSF at room temperature (because luciferase is not stable at higher temperatures). Calcium was uncaged and imaged from single astrocytes as described above, using the red Ca^2+^ indicator Rhod-2 (50 μM) instead of Fluo-4. A field of view containing an astrocyte and its processes was selected and ATP-derived bioluminescence was collected in darkness using highly sensitive GaAsP detectors with a Zeiss LSM780 2-photon/confocal microscope.

### Estimating axonal conduction velocity

We estimated axonal conduction velocities based on parameters acquired experimentally from dual soma and axon bleb patch-clamp recordings, live confocal imaging and *post-hoc* immunostaining of layer V pyramidal neurons. In accordance with previous studies, two assumptions were made: (i) spikes initiate at the distal half of the AIS (the AIS being defined as being from the edge of the soma to the start of the first Caspr-labeled paranode) because in the distal AIS Na^+^ channels are found in high density, have a low voltage threshold and are isolated from the capacitive load of the soma (44, 45); (ii) forward conduction speed is 2-3 times faster than backward conduction speed because of the lower Na^+^ channel density in the proximal AIS and the capacitive load of the soma (16, 45). The axonal conduction speed can be then calculated as follows:

(3) $$v=\frac{x_{bleb}-2x_{soma}}{T}\;or\;v=\frac{x_{bleb}-3x_{soma}}{T}$$

where *v* is the speed from the spike initiation site to the axon bleb, *T* is the delay time from the somatic signal to the bleb signal (obtained from dual patch-clamp recordings), and *x_beb_* and *x_soma_* are the distances from the spike initiation site to the recording sites on the axon bleb or on the soma, respectively (obtained from confocal imaging by assuming a position for spike initiation).

### Computer simulations of infinite corpus callosal axon

**Fig. 3:**
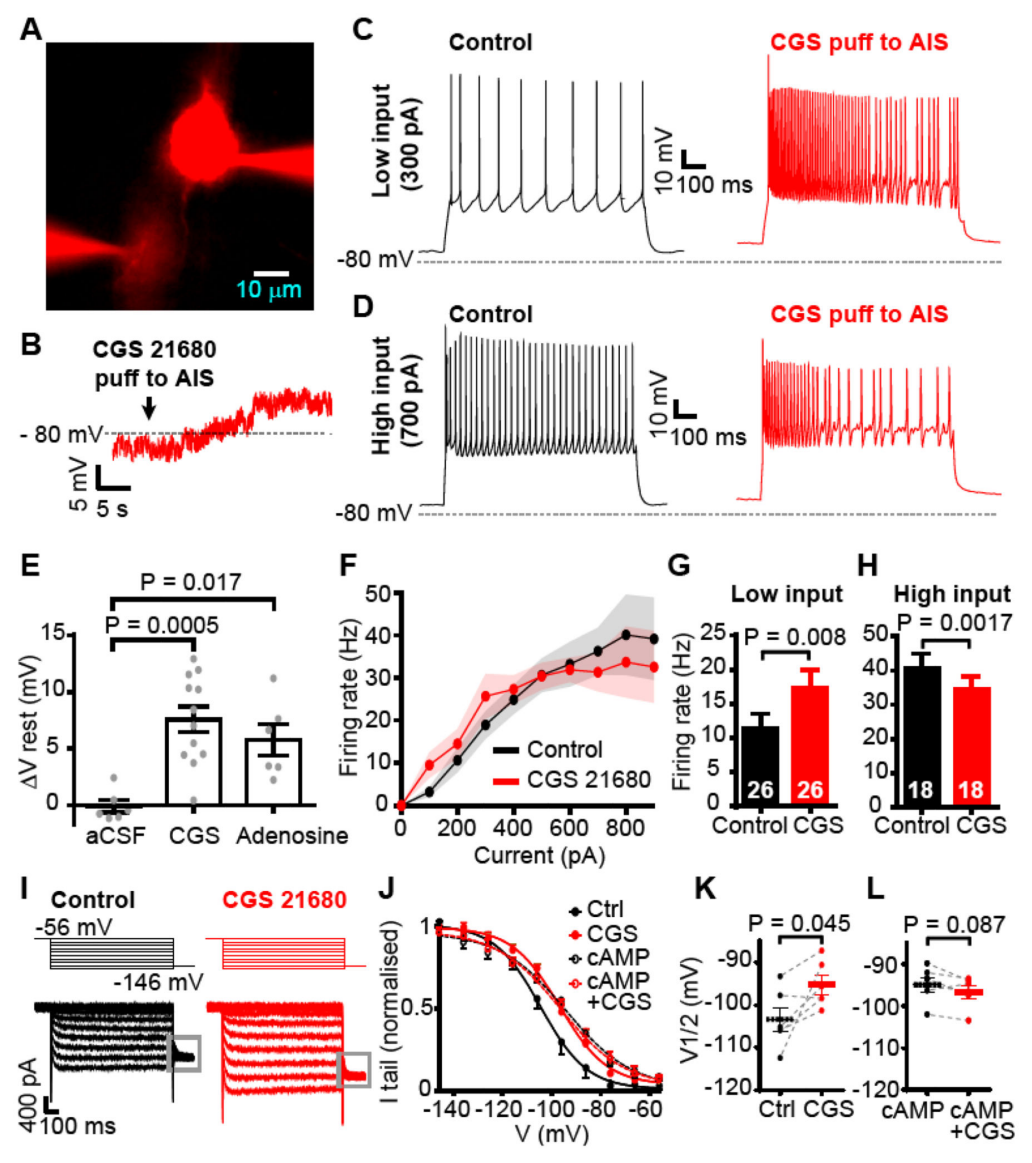
A_2a_ receptors, via cAMP and I_h_, modulate excitability at the AIS. (**A**) Patch-clamped layer V pyramidal cells loaded with Alexa 594. Lower pipette puffing adenosine at distal AIS contains Alexa 594 to delineate region affected by adenosine. (**B**) Depolarization of soma evoked by puffing the A_2a_R agonist CGS 21680 (0.5 μM). (**C-D**) Voltage response to (**C**) 300 pA or (**D**) 700 pA injected current with and without CGS 21680 application. (**E**) Mean resting potential change when puffing aCSF (n=6), 0.5 μM CGS 21680 (n=12) or 100 μM adenosine (n=6) onto the AIS. (**F**) Firing rate averaged over 1 sec as a function of injected current, in control conditions or with CGS 21680 applied to the AIS (n=9; s.e.m. shown faint). (**G-H**) Firing rate change for (**G**) “low” (averaged over 100-300 pA, 26 current steps from 9 cells) or (**H**) “high” (700-900 pA, 18 current steps from 8 cells) input currents. (**I**) Specimen currents on stepping from -56 mV to various pulse potentials, and then to -136 mV to evoke tail currents allowing construction of the activation curve, in control conditions and with CGS 21680 puffed at the AIS. (**J**) I_h_ activation curves for normal conditions, puff application of CGS 21680 (n=6), and both of these with 50 μM cAMP included in the patch pipette (n=6). (**K**) V_1/2_ values (50% activation voltage of fitted Boltzmann curve) before (Ctrl) and during CGS 21680 application. (**L**) As in K, but with cAMP in patch pipette. All data are from rat.

As in our previous studies (17, 46, 47), action potential conduction along myelinated axons was simulated using MATLAB. Electrophysiological parameters were based on the finite impedance double cable model (model C) of Richardson et al. (48), except that the membrane capacitance was taken as the physiologically measured value of 0.9 μF/cm^2^ (49). The differential equations of the model were derived and solved as in the myelinated axon model of Halter and Clark (50), in which the axon is divided into compartments representing the node, paranode and internode. The MATLAB code including all the parameters and equations is available from Ref. (18). Conduction speed simulations of long callosal-like axons were carried out as previously (17), where the parameters used were based on experimental data. The speed was measured between nodes 20 and 30 in a uniform axon containing 51 nodes and 50 internodes of constant lengths (nodes: 1.5 μm, internodes: 81.7 μm) and diameters (nodes: 0.64 μm, internodes: 0.73 μm). The periaxonal space thickness, which has an important effect on conduction speed (17, 51), was set at 15 nm, except in the paranodes (the 1.9 μm end parts of the internodes) where the width was reduced to 0.0123 nm (17). Assuming a myelin wrap periodicity of 15.6 nm, 5 myelin wraps were needed to set the g-ratio close to 0.8. Each node expressed fast Na_V_, persistent Na_V_ and slow K_V_ channels at fully-activated conductance densities of 10, 0.01 and 0.4 mS/mm^2^ respectively, as well as adenosine-gated I_h_ channels at a density of either 0 (for no A_2a_R activation) or 0.1565 (to mimic A_2a_R activation) mS/mm^2^. This density was experimentally derived as follows: the maximum I_h_ conductance (derived from tail current analysis as in Fig. 3I-J) evoked in the ~10 μm length of the distal AIS was divided by the area of the distal AIS to obtain a fully-activated conductance density of 0.111 mS/mm^2^. Immunohistochemical labeling of A_2a_Rs then showed their density at nodes of Ranvier to be a factor of 1.41 higher than in the distal AIS (calculated by integrating the background-subtracted fluorescent signal across a small length w of the axon and dividing by π.w.d, where d is the diameter of the axon), implying a density at the nodes of 0.1565 mS/mm^2^. The node resting potential in the absence of adenosine was set to -82 mV by adjusting the magnitude of a leak conductance (to 0.113 mS/mm^2^) with a reversal potential of -84 mV (the reversal potential of the other K^+^ currents in the model). The leak current represents K^+^ leak channels such as TRAAK present in the nodes of Ranvier (52, 53).

### Computer simulations of patch-clamped neuron with soma, AIS and 3 internodes

**Fig. 4:**
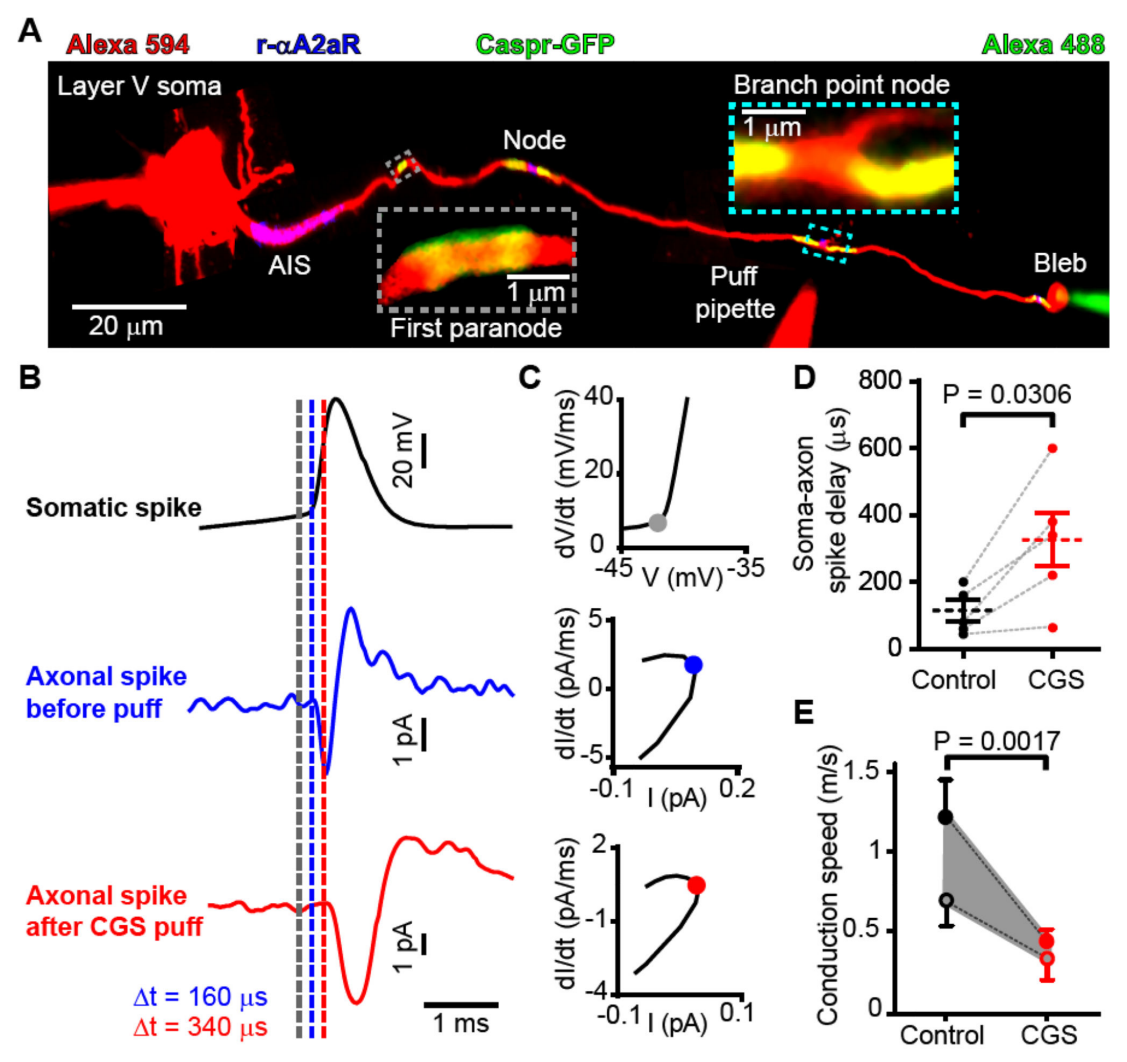
A_2a_ receptors in the node of Ranvier modulate conduction velocity. (**A**) Myelinated axon in Thy1-Caspr-GFP mouse filled with Alexa 594 and patch-clamped at the cell soma and end-of-axon bleb. (**B**) Average of >100 evoked action potentials in the soma and bleb in control conditions and while puffing 0.5 μM CGS 21680 at a node of Ranvier. Dashed lines show times of initiation of action potential derived from threshold values of dV/dt and dI/dt (see text). (**C**) Phase plane plots showing times indicated on B (dots). (**D**) Response latency in bleb. (**E**) Conduction velocities derived making assumptions discussed in the main text (closed circles assume spike starts at the middle of the AIS and the forward speed is twice the backward speed; open circles assume spike starts at the end of the AIS and the forward speed is three times faster than the backward speed). Data in A-C are from mouse; data in D-E combine data from rats and mice (neither the initial speed nor the percentage change evoked by CGS 21680 differed significantly between rats and mice, p=0.43 and 0.15 respectively).

**Fig. 5:**
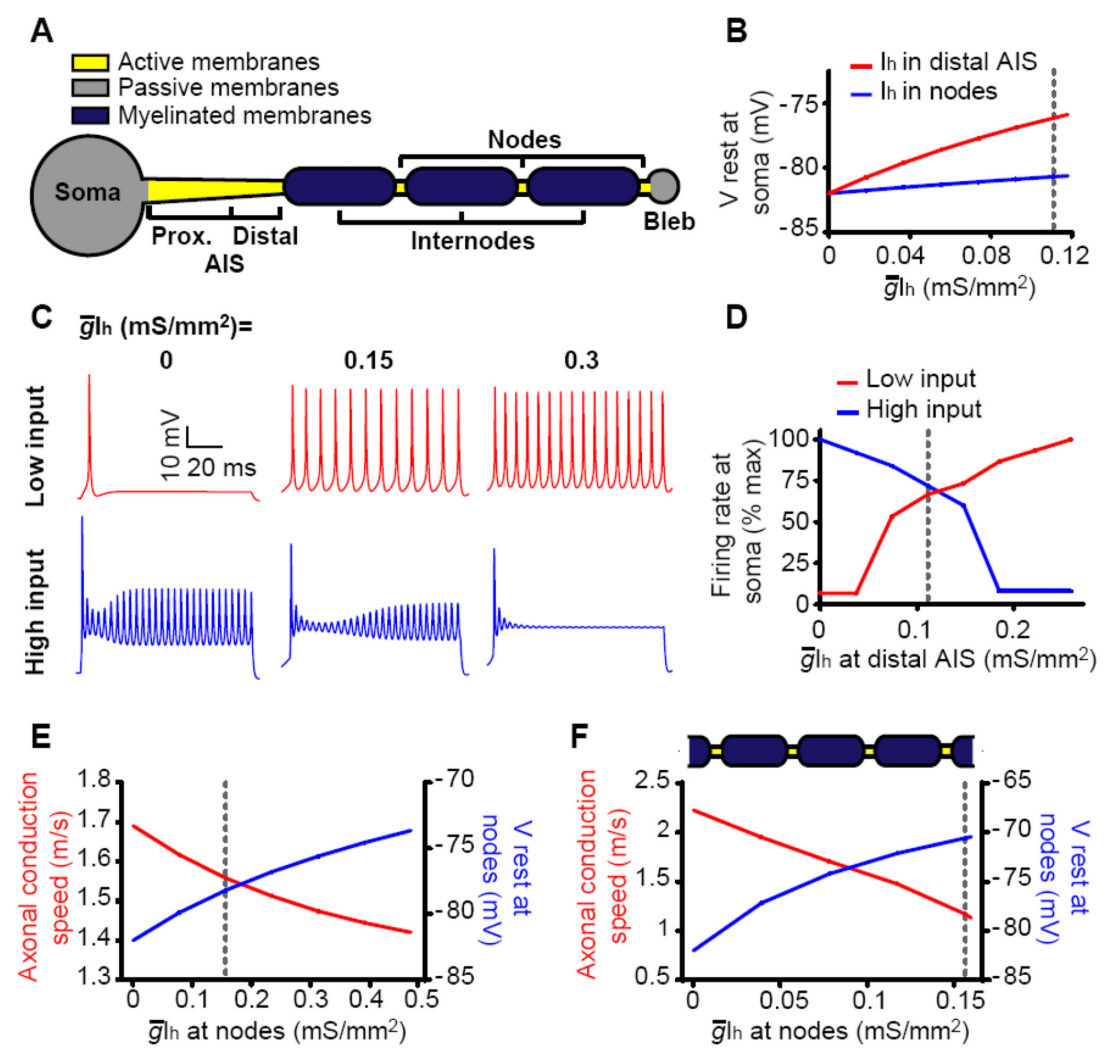
Computational modeling predicts the adenosine-evoked decrease of axonal conduction speed. (**A**) Schematic diagram of the model of the experiment in Fig. 4. (**B**) Adding different densities of adenosine-sensitive maximal I_h_ conductance $\left({\bar{\text{g}}\text{I}}_{\text{h}}\right)$ to the distal AIS evokes a larger soma depolarization than adding it to the nodes of Ranvier. Vertical dashed line shows measured maximal conductance (0.11 mS/mm^2^) (**C**) Voltage response at soma to injecting 20 pA (top row) or 180 pA (bottom row) with 3 different levels of ${\bar{\text{g}}\text{I}}_{\text{h}}$ added (as indicated) to the distal AIS. As observed experimentally (Fig. 3C-H) at low injected current the action potential frequency is increased; at high injected current it is decreased (owing to the simulated decrease of action potential amplitude, we defined an action potential as occurring if the voltage crossed -50 mV). (**D**) Firing rate as a function of ${\bar{\text{g}}\text{I}}_{\text{h}}$ for simulations as in C. (**E**) Action potential speed from the first to the last node in A, and average resting potential of the 3 nodes, as a function of ${\bar{\text{g}}\text{I}}_{\text{h}}$ added to each node (vertical dashed line shows the estimated physiological value of 0.1565 mS/mm^2^, see Materials and Methods). (**F**) Predictions of infinite axon model for conduction speed and node resting potential as a function of ${\bar{\text{g}}\text{I}}_{\text{h}}$.

**Fig. 6:**
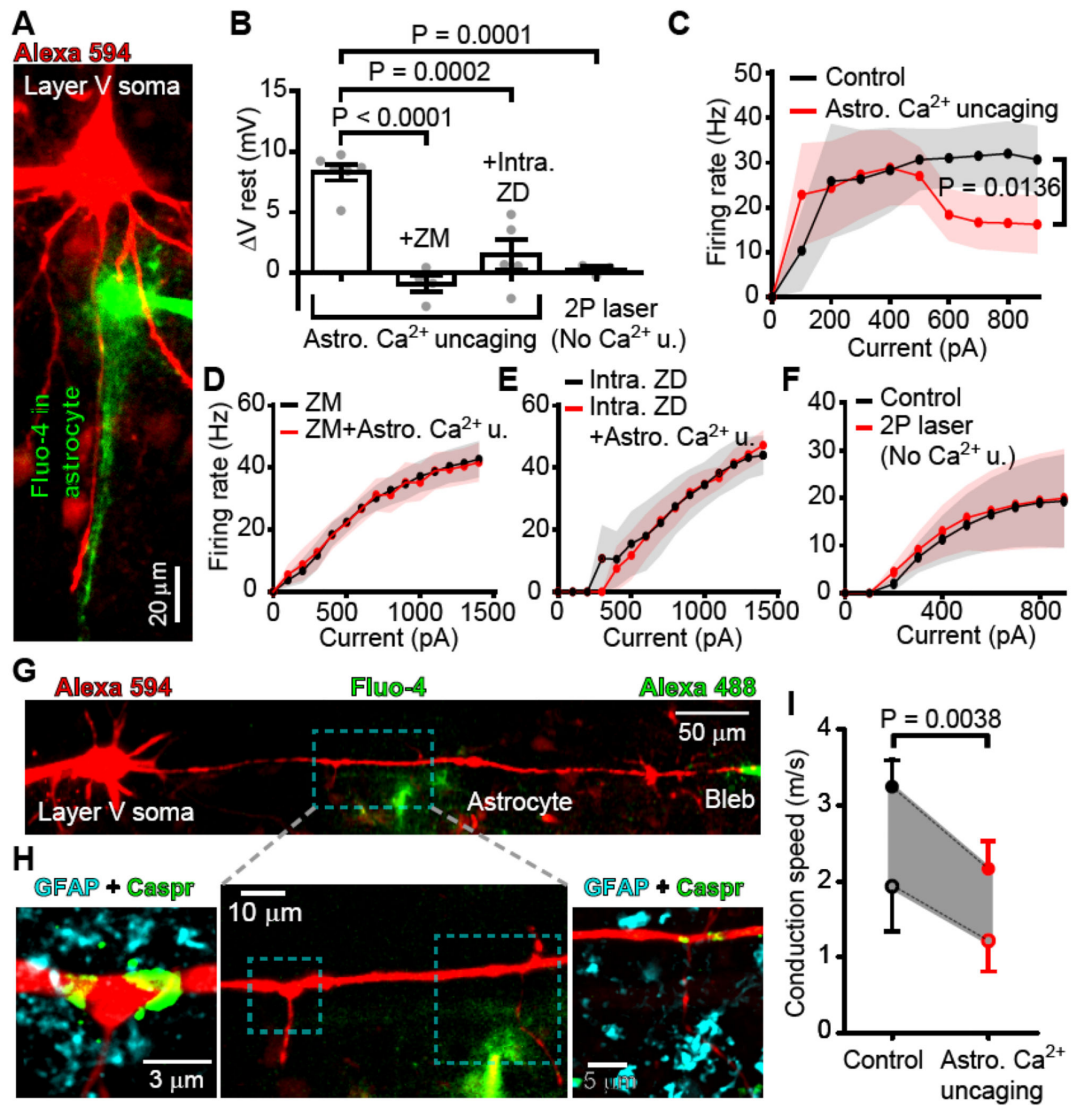
Ca^2+^ concentration rises in astrocytes regulate pyramidal cell excitability and axonal conduction speed. (**A**) A patch-clamped L5 pyramidal neuron (with Alexa 594 in the left pipette, red) and a periaxonal astrocyte filled with the Ca^2+^ cage NP-EGTA and Fluo-4 (right pipette, green). [Ca^2+^]_i_ rises in astrocyte processes near the axon (see movie S3). (**B**) Resting potential (Vrest) depolarized following astrocyte Ca^2+^ uncaging, but not when blocking A_2a_Rs with superfused ZM 241385 (100 nM) or I_h_ channels with ZD7288 (20 μM in the pipette), nor when 2-photon laser excitation failed to raise [Ca^2+^]_i_ (No Ca^2+^ u.; see Fig. S2) (Astro. Ca^2+^ uncaging: n=6, +ZM: n=4, +Intra. ZD: n=5, 2P laser without uncaging: n=3; one-way ANOVA p<0.0001). (**C**) Neuronal firing rate evoked by injecting 1 s current steps in 100 pA increments before (black) and after astrocytic Ca^2+^ uncaging (red). (**D-F**) As in C but with ZM 241385 (**D**, n=4), or ZD7288 (**E**, n=4), or with illumination that failed to uncage Ca^2+^ (**F**, n=3). (**G**) Live imaging of a L5 pyramidal neuron patch-clamped at the soma (left pipette, loading red Alexa 594) and the axon end (right pipette, green). A perinodal astrocyte (near axon branches) was patch-filled with NP-EGTA and Fluo-4. (**H**) Middle: high resolution image of the dashed box in G. Left and right images show the areas in the dashed boxes of the middle image, after immunostaining for GFAP and Caspr. Nodes flanked by Caspr (green) are close to GFAP-positive astrocyte processes (cyan). (**I**) Estimated axonal conduction speed before and after astrocyte Ca^2+^ uncaging (see text associated with Fig. 4E for assumptions made). Data in panels A-H are from rat; panel I combines data from rats and mice (neither the initial speed nor the percentage change evoked by Ca^2+^ uncaging differed significantly between rats and mice, p=0.08 and 0.93 respectively).

This model was then modified so that the dimensions of a specimen patch-clamped and imaged neuron acquired for the present study (that in Fig. 4) were implemented (Fig. 5A). The model included “nodes” representing the soma, the proximal AIS, the distal AIS, 3 nodes of Ranvier and a terminating bleb. To represent the area and thus the capacitance of the real soma, the diameter and length of the cylindrical “node” representing the soma were both set to 18 μm. The proximal and distal AIS had lengths of 21.9 and 10 μm respectively, and diameters (measured experimentally as the average over each of these two zones) of 1.046 and 0.525 μm respectively. The soma and proximal AIS, and the proximal and distal AIS, were separated by extremely small (10^-7^ μm long) “internodes” (which are essential for the alternating node-internode model to function in MATLAB; not shown in Fig. 5A) with diameters of 1.046 and 0.79 μm, respectively. Distal to the AIS are 3 internodes of lengths 24.91, 52.44 and 39.20 μm and diameters 0.51, 0.56 and 0.53 μm respectively, each followed by nodes of Ranvier of lengths 1.126, 0.915 and 0.414 μm, and diameters 0.445, 0.452 and 0.401 μm, respectively. The 3rd node of Ranvier was followed by an extremely small (10^-7^ μm long) “internode” of diameter 0.4 μm, and then a terminal bleb of length 1.64 μm and diameter 3 μm. Each node of Ranvier expressed the same conductances as in the infinite cable model. The proximal and distal AIS compartments expressed fast Na_V_, persistent Na_V_ and slow K_V_ channels at the same densities as in the infinite cable model, and in addition a low threshold K_V_ channel with a maximum conductance of 0.4 mS/mm^2^ which was needed to allow the generation of repetitive action potential trains in response to current injected at the soma. Adenosine insensitive I_h_ conductance (with a half-maximum activation voltage of -103.5 mV, as found on average in experiments as in Fig. 3J) was expressed in the soma and proximal AIS (as seen immunohistochemically for HCN1 channels) at a density of 0.0033 mS/mm^2^ (set so as to reproduce the mean I_h_ maximal conductance seen in the absence of adenosine in Fig. 3I, J), while adenosine-sensitive I_h_ was present in the distal AIS at a density of 0.111 mS/mm^2^ as well as in the nodes of Ranvier at a density 1.41-fold higher (see above). The density used for the distal AIS was set to increase the maximum I_h_ conductance measured at the soma by 51% as observed during activation of A_2a_Rs in the distal AIS (Fig. 3I, J) and the midpoint of its activation curve was at -80 mV to reproduce the positive shift for the total I_h_ apparent activation curve in Fig. 3J. The terminal bleb expressed no voltage-gated conductances. To match the model to the experimental data we wanted to reproduce the 5.8 mV depolarization recorded at the soma when puffed adenosine activated I_h_ in the distal AIS (Fig. 3E). We found that if we set the resting potential to -82 mV in the soma and AIS using a leak conductance with a reversal potential of -84 mV (as in the nodes), then this introduced too much resting conductance, reducing the depolarization evoked at the soma when A_2a_Rs were activated in the distal AIS to only +1.55 mV. Accordingly, in the soma, AIS and terminal bleb, we used a nominal leak reversal potential of -4000 mV, converting the leak into an essentially voltage-independent (~zero conductance) current, similar conceptually to the constant outward current generated by the Na^+^/K^+^ pump. The adenosine-sensitive I_h_ (when activated in the distal AIS) then generated a depolarization of 5.9 mV at the soma. To investigate the effect of the adenosine-evoked I_h_ current, the action potential speed was measured between the first and the last nodes of Ranvier when current was injected briefly (1 nA for 100 μsec) at the soma. The model reproduced reasonably well the assumptions we made to assess the conduction speed in our experiments: in the simulations, spikes evoked by current injection at the soma were initiated in the distal AIS “node”, the forward conduction speed was faster than the backward speed (from distal AIS to bleb: 1.33 m/s; from distal AIS to soma: 0.23 m/s) and the conduction speed from the distal AIS to the bleb (1.33 m/s) was in the range estimated from the experiments in Fig. 4E and 6I (1.31-2.23 m/s).

### Statistics

Statistical analyses and graphs were performed with GraphPad Prism 6. Data are presented as mean ± SEM. Data normality was assessed with D’Agostino & Pearson omnibus or Kolmogorov-Smirnov tests. Comparisons of normally distributed two groups were made using two-tailed Student *t*-tests. When treatments involved more than two independent groups, statistical comparisons were performed with one-way ANOVA and *post hoc* Tukey’s multiple comparison. Data that were not normally distributed were analysed with Mann-Whitney test or Wilcoxon matched-pairs signed rank test. Assessment of whether the slope of linear regressions differed significantly from zero was obtained using the *t*-statistic for the slope. P-values within any figure panel were adjusted for multiple comparisons.

### Gender and species effects

Mice were used in experiments when it was essential to have the nodes labelled with Caspr-GFP. Rats were also used because they are more generally available at all ages in our university. We observed no differences between the two species, for example in:

(i) the presence of A_2a_Rs and HCN channels at nodes of Ranvier and the AIS;
(ii) the initial speed and the percentage change evoked by CGS 21680 puff onto nodes, which did not differ significantly between rats and mice (p=0.43 and 0.15 respectively);
(iii) the initial speed and the percentage change evoked by Ca^2+^ uncaging in perinodal astrocytes, which did not differ significantly between rats and mice (p=0.08 and 0.93 respectively).

Animals of both sexes were used because of increasing awareness of the fact that gender can play a role in determining function. For major results, we found no differences between the two sexes, for example in:

(i) the presence of A_2a_Rs and HCN channels at nodes of Ranvier and the AIS;
(ii) the initial resting membrane potential and the change evoked by CGS 21680 puff onto the AIS, which did not differ significantly between males and females (p=0.69 and 0.75 respectively);
(iii) the initial speed and the percentage change evoked by CGS 21680 puff onto nodes, which did not differ significantly between males and females (p=0.3 and 0.45 respectively).

## Supplementary Material

Supplementary movies

Supplementary Material

## Figures and Tables

**Fig. 1:**
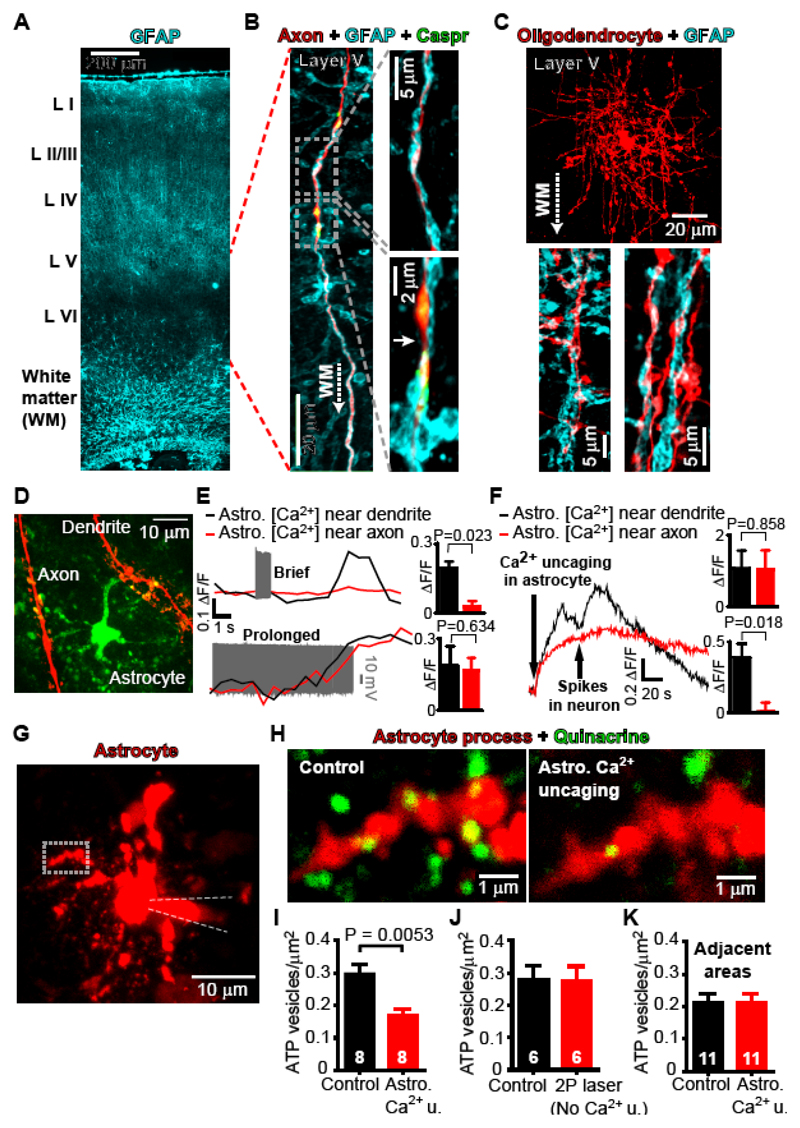
Astrocyte processes near myelinated axons release ATP in response to [Ca^2+^]_i_ rises. (**A**) GFAP labeling of mouse coronal slice shows astrocytes in grey and white matter. (**B**) Patch-clamp loaded Alexa 594 labels axon of layer V pyramidal cell. Expanded internodal (top) and nodal (bottom, identified by Caspr labeling, node is at arrow) regions reveal astrocytes at both locations. (**C**) Patch-clamped oligodendrocyte in layer V with insets below showing GFAP labeling around myelinated internodes. (**D**) Dendrite and axon of patch-clamped rat layer V pyramidal cell near an astrocyte loaded with Ca^2+^ sensor Fluo-4. (**E**) [Ca^2^]i response in astrocyte processes near dendrite (black) and axon (red) to neuron depolarization with 500 pA for 1 sec (top, n=5) or 10 sec (bottom, n=3) to evoke spiking (grey). (**F**) [Ca^2+^]_i_ response in astrocyte processes to Ca^2+^ uncaging and to brief neuronal spike trains (n=6). Top bar chart shows Ca^2+^ response just before spiking; bottom bar chart shows Ca^2+^ response following neuron spiking evoked by 500 pA for 1 sec. (**G**) Patch-clamped rat astrocyte loaded with Ca^2+^ cage NP-EGTA and Rhod-2 to measure [Ca^2^]_i_ showing region imaged for H. (**H**) Quinacrine labeled puncta in astrocyte process are depleted on uncaging Ca^2+^ (Rhod 2 may not enter the smallest astrocyte processes, explaining why some puncta appear outside the astrocyte). (**I-K**) Quantification of ATP vesicles present per μm^2^ of astrocyte process: (I) before and after uncaging (Astro. Ca^2+^ u.); (J) before and after excitation that did not evoke a [Ca^2^]i rise in the astrocyte (No Ca^2+^ u.; see Fig. S2); (**K**) in regions outside the astrocyte (5 μm away) before and after uncaging. Numbers of processes shown on bars. Processes came from 12 cells. Panels A-C are on mice, D-K are on rats.

**Fig. 2:**
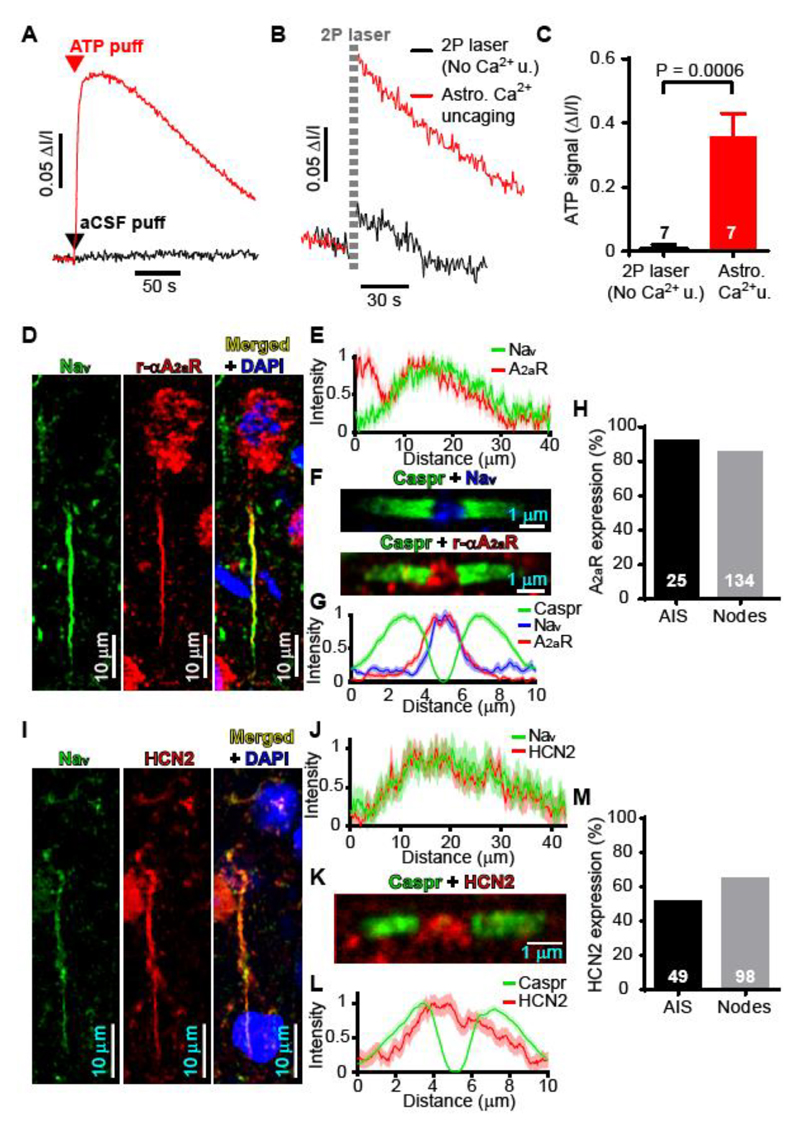
ATP release from astrocytes may target adenosine receptors on myelinated axons of layer V pyramidal neurons. (**A**) Using luciferin-luciferase to detect ATP puffed into extracellular solution. (**B**) Response to 2-photon excitation uncaging Ca^2+^ in astrocytes evoked a luciferin-luciferase signal, unless the excitation failed to raise astrocyte [Ca^2+^]i (No Ca^2+^ u.; see Fig. S2). (**C**) Quantification of experiments in B in 7 cells. (**D-H**) A_2a_Rs are present in the AIS (**D**) where they overlap with voltage-gated Na^+^ channel (Na_v_) expression (**E**, mean of 14 Na_v_ and 25 A_2a_R profiles) and at the node of Ranvier (**F**) where they overlap with Na_v_ and are flanked by Caspr labeling (**G**, mean of 48 Caspr, 48 A_2a_R and 24 Na_v_ profiles). (**H**) Percentage of 25 AISs and 134 nodes that express A_2a_Rs. (**I-M**) HCN2 channel subunits are present in the AIS (**I**) where they overlap with voltage-gated Na^+^ channel (Na_v_) expression (**J**, mean of 19 Na_V_ and 16 HCN2 profiles) and at the node of Ranvier (**K**) where they overlap with Na_v_ and are flanked by Caspr labeling (**L**, mean of 53 Caspr and HCN2 profiles). (**M**) Percentage of 49 AISs and 98 nodes that express HCN2. Panels A-E and I-J are on rats; F-G and K-L are on mouse; H and M combine rat AIS and mouse node data.

## Data Availability

All data are available in the manuscript or the supplementary materials. Thy1-Caspr-GFP mice are available under a Material Transfer Agreement with the University of Edinburgh from Peter Brophy and Diane Sherman. Code used for simulations is freely available (54). This research was funded in part by grants 099222/Z/12/Z and 219366/Z/19/Z from the Wellcome Trust, a cOAlition S organization. The authors will make the Author Accepted Manuscript (AAM) version available under a CC BY public copyright license.

## One sentence summary

Astrocytes control myelinated axon speed

## Code availability

The computer code used for the simulations described above has been deposited in Zenodo (54).
