# Supplementary material for: Astrocyte Ca2+-evoked ATP release regulates myelinated axon excitability and conduction speed
Source: Science. Author manuscript; Available in PMC 2021 Nov 9. (PMC7611967; doi:10.1126/science.abh2858)
Supplement: Supplementary movies [file EMS137883-supplement-Supplementary_movies.zip › science.abh2858_movie_captions.pdf]

Supplementary movie captions

Movie S1. Neuronal activity triggers Ca^{2+} transients in astrocyte processes near axons and dendrites. An astrocyte was loaded with Fluo-4 to monitor its Ca^{2+} activity following neuron stimulation. The astrocyte processes contact the axon (left) and a dendrite (right) of a patch-clamped neuron dye-filled with Alexa 594 (same astrocyte as in Fig. 1D). In this experiment, robust firing of the neuron triggered Ca^{2+} transients in processes contacting the dendrite and the axon. Fluorescence at baseline was subtracted from all the time-lapse images.

Movie S2. Ca^{2+} uncaging in a periaxonal astrocyte. A L5 pyramidal neuron was whole cell patch-clamped and dye-filled with Alexa 594 (loaded in the left pipette, red), and an astrocyte (green) was loaded with NP-EGTA and Fluo-4 to uncage Ca^{2+} and monitor its level. Ca^{2+} was uncaged with 2-photon excitation at 720 nm targeted at the astrocyte soma. This led to a $[Ca^{2+}]_i$ rise in astrocyte processes, including those contacting the axon. Time-lapse images of the astrocyte were superimposed on a still image of the neuron to highlight the position of the astrocyte and its processes with regard to the axon. In this experiment, neuronal activity was monitored before and after astrocyte Ca^{2+} uncaging.

Movie S3. Astrocyte Ca^{2+} propagates in processes near the axon. Time-lapse images of the astrocyte shown in Fig. 6A. Ca^{2+} was uncaged at the soma by 2-photon excitation and is seen propagating in the processes going downwards, following the same direction of the axon of the L5 pyramidal neuron, towards the callosal white matter.
