# Supplementary material for: Astrocyte Ca2+-evoked ATP release regulates myelinated axon excitability and conduction speed
Source: Science. Author manuscript; Available in PMC 2021 Nov 9. (PMC7611967; doi:10.1126/science.abh2858)
Supplement: Supplementary Material [file EMS137883-supplement-Supplementary_Material.pdf]

Supplementary Materials for

Astrocyte Ca^{2+}-evoked ATP release regulates myelinated axon excitability and conduction speed

Jonathan Lezmy, Lorena Arancibia-Carcamo, Tania Quintela-Lopez, Diane L. Sherman, Peter J. Brophy and David Attwell

Correspondence to: j.lezmy@ucl.ac.uk and d.attwell@ucl.ac.uk

This PDF file includes:

Figs. S1 to S7
Captions for Movies S1 to S3

Other Supplementary Materials for this manuscript include the following:

Movies S1 to S3
MDAR Reproducibility Checklist

Supplementary figures and captions

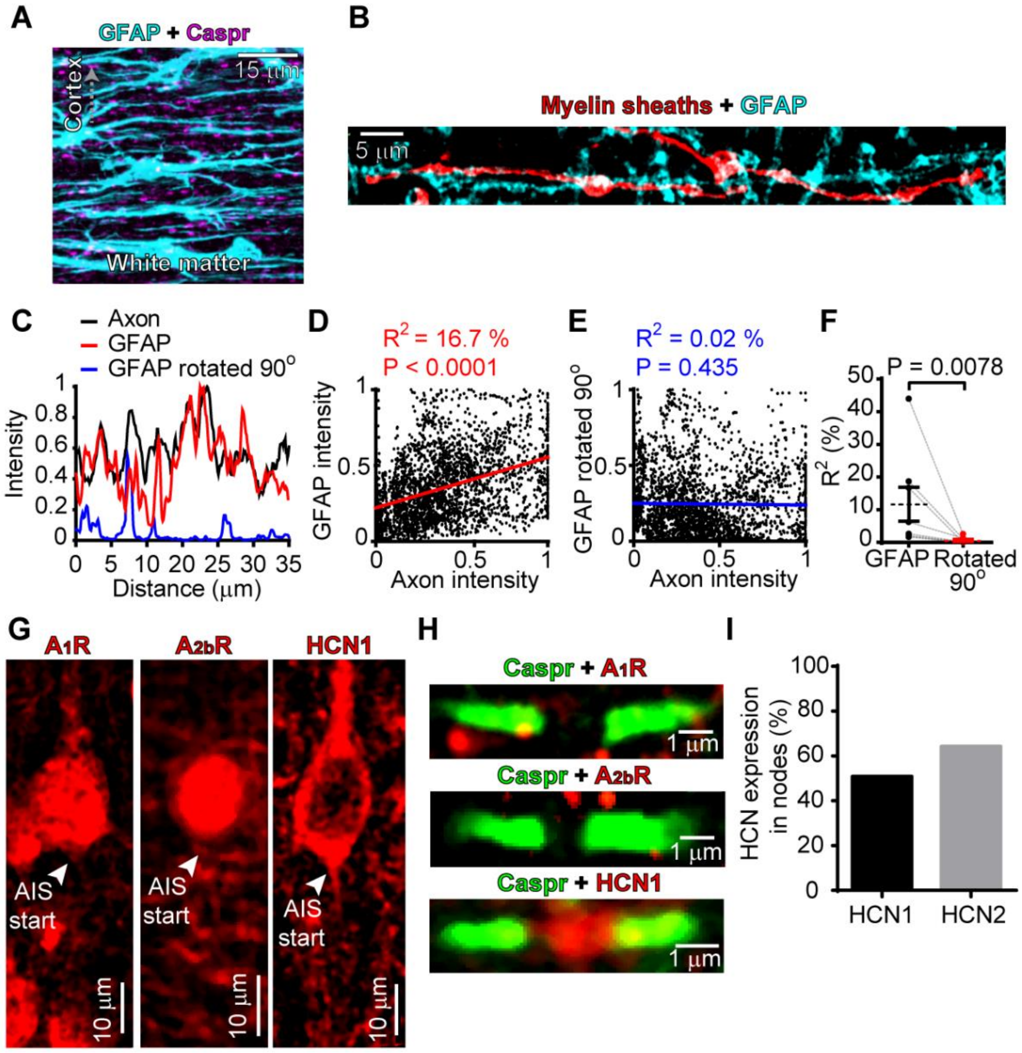

Fig. S1. Astrocyte association with myelinated axons and HCN labeling. (**A**) GFAP labeling (cyan) in the corpus callosum of the Thy1-Caspr-GFP mouse in which paranodes are labeled (magenta). Astrocyte processes run parallel to the axons. (**B**) Example of myelinated internodes of dye-filled oligodendrocyte and its associated astrocyte processes. (**C**) Intensities (arbitrary units) of axon (black) and astrocyte labeling (red) along the axon averaged over a 5 µm wide band centred on the axon. Blue line shows result obtained after rotating the GFAP image though 90°. (**D**) Scatterplot of integrated GFAP and axon dye intensities for the data in C shows correlation of the data (r^2=0.17, slope is significantly non-zero with p<0.0001). (**E**) As for D but with GFAP image rotated 90° (r^2=0.0002, slope is not significantly different from zero with p=0.435). (**F**) Correlation coefficients from plots like D and E for 3 neurons and 3 oligodendrocytes. P-value from Wilcoxon test. (**G**) Three different layer V somata immunolabeled for A_1 and A_{2b} receptors, and for HCN1 channel subunits. (**H**) Three nodes labeled for Caspr and A_1, A_{2b} receptors or HCN1. (**I**) Percentage of nodes showing labeling for HCN1 and HCN2. Although the fraction of nodes expressing HCN subunits appears lower than for A_{2a} receptors (Fig. 2), the high success rate of our electrophysiological experiments implies that HCN expression is actually higher than the numbers detected with immunohistochemistry, as reported previously (*15*). All data are from mouse.

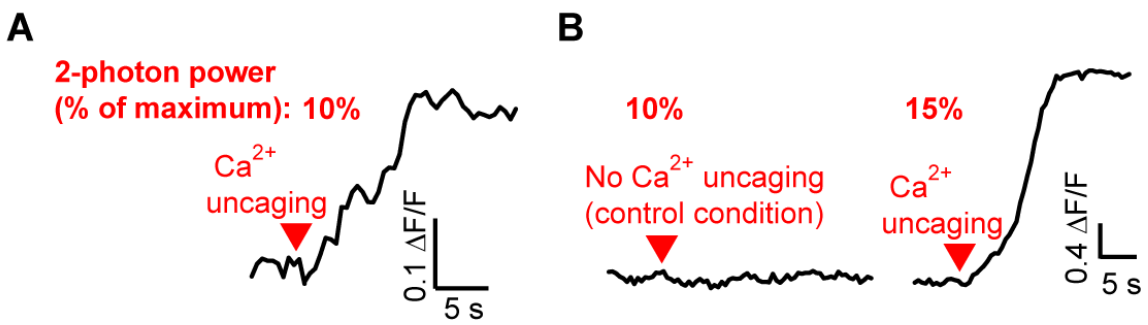

Fig. S2. Dependence of [Ca^{2+}]$_i$ rise evoked by uncaging on illumination intensity. (**A**) Specimen [Ca^{2+}]$_i$ response in an astrocyte soma following 2-photon illumination at 10% maximum power. (**B**) Specimen [Ca^{2+}]$_i$ response in another astrocyte. The same laser power as in A (10%) did not elicit a detectable [Ca^{2+}]$_i$ rise in this astrocyte soma, but increasing the 2-photon laser power to 15% raised the [Ca^{2+}]$_i$. The non-linear dependence of the [Ca^{2+}]$_i$ rise on illumination intensity may indicate a contribution of Ca^{2+}-induced Ca^{2+} release to the uncaging-evoked [Ca^{2+}]$_i$ rise. All data are from rat.

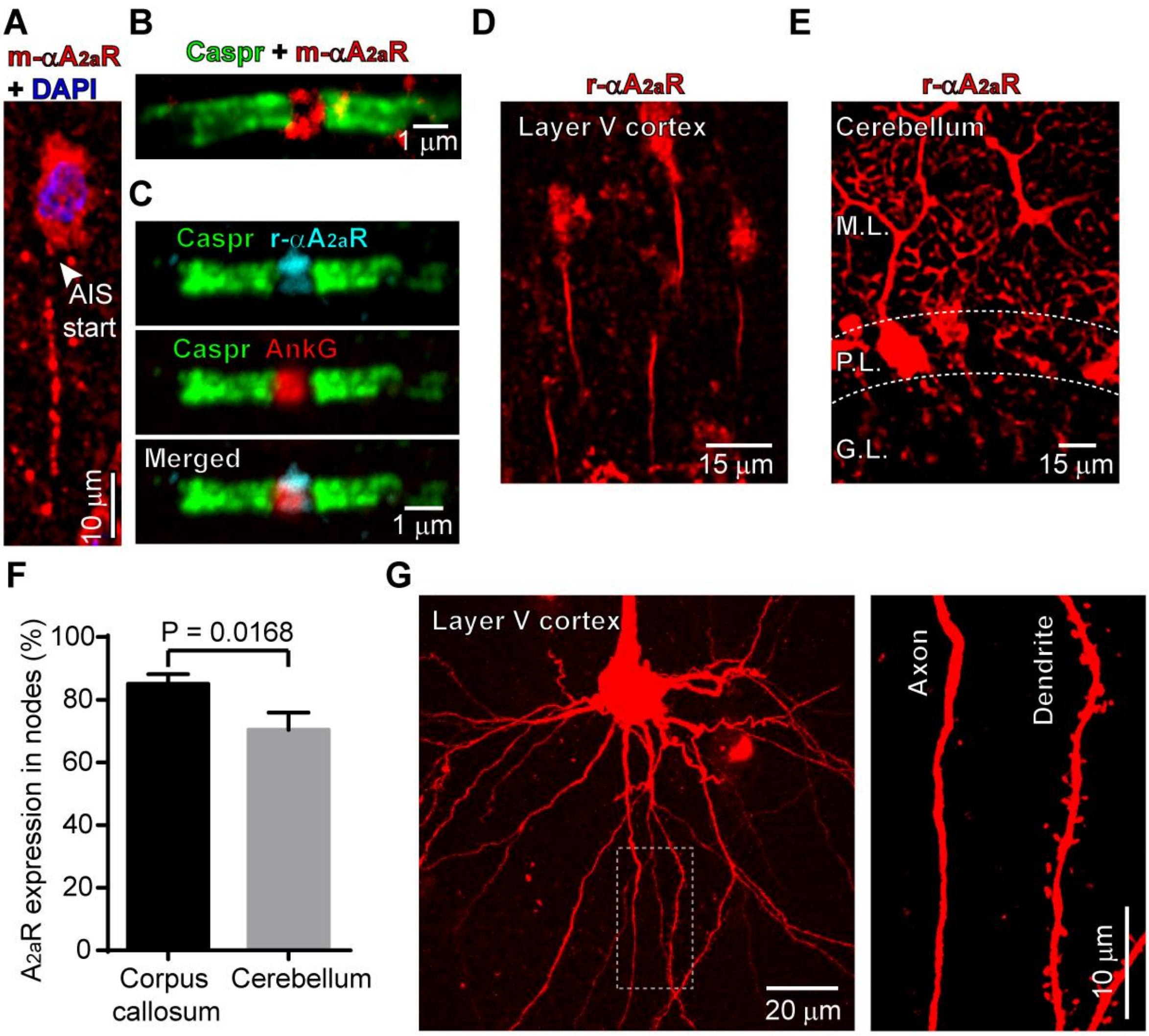

Fig. S3. $A_{2a}R$ localisation in the cortex and the cerebellum. (**A**, **B**) Labelling of $A_{2a}R$ in an AIS and a node of Ranvier with a mouse monoclonal antibody previously tested on $A_{2a}R$-KO tissue. (**C**) Co-localisation of $A_{2a}R$ and the structural protein Ankyrin G in a node. (**D**, **E**) Overview of $A_{2a}R$ expression (using rat antibody) in cortex layer V (**D**) and in the molecular (M.L.), Purkinje (P.L.) and granular (G.L.) layers of the cerebellum (**E**). $A_{2a}R$ was not detected in the AISs of Purkinje cells. (**F**) Percentage of 134 and 71 nodes that express $A_{2a}R$ in the corpus callosum and the cerebellar white matter, respectively. P-value from Mann-Whitney test. (**G**) Specimen layer V pyramidal neuron dye-filled with Alexa 594 with a large apical dendrite going towards the layer I and a spineless axon emerging from the base of the soma going towards the corpus callosum. High resolution image of the dashed box region (right) shows the spines on a dendrite near the axon (some processes are absent from the high resolution image compared to the left panel because a different stack depth was used). Panels A, D, E and G are from rat; B-C and F are from mouse.

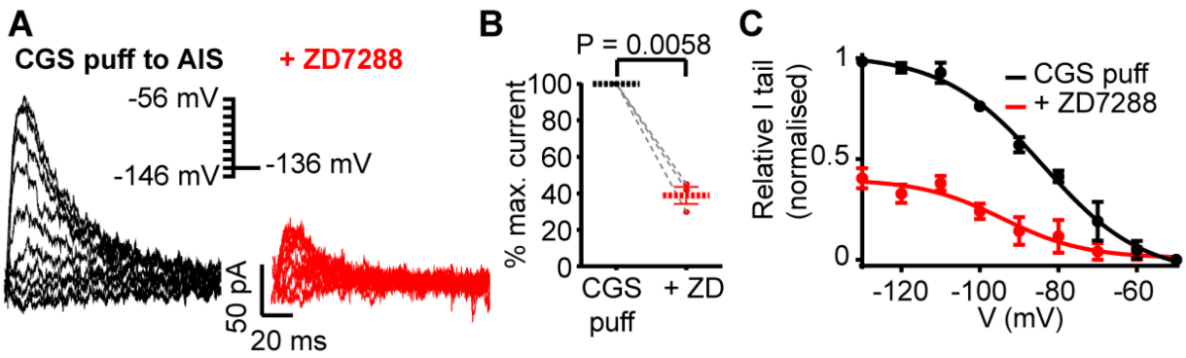

Fig. S4. HCN (I_h) subunits generates the tail currents seen at -136 mV. (**A**) Tail currents at -136 mV after steps to different potentials following a CGS puff to the AIS (left) and after the HCN blocker ZD7288 (50 μM) was bath applied (right). (**B**) Mean data showing a 61% reduction in maximum current in ZD7288 (CGS puff: 100 %, +ZD7288: 38.9±4.7%; p=0.0058, paired *t*-test, n=3). (**C**) I_h activation curves generated from the tail currents. Activation curve with ZD7288 was normalised to the maximum current in control conditions to highlight the effect of blocking HCN. All data are from rat.

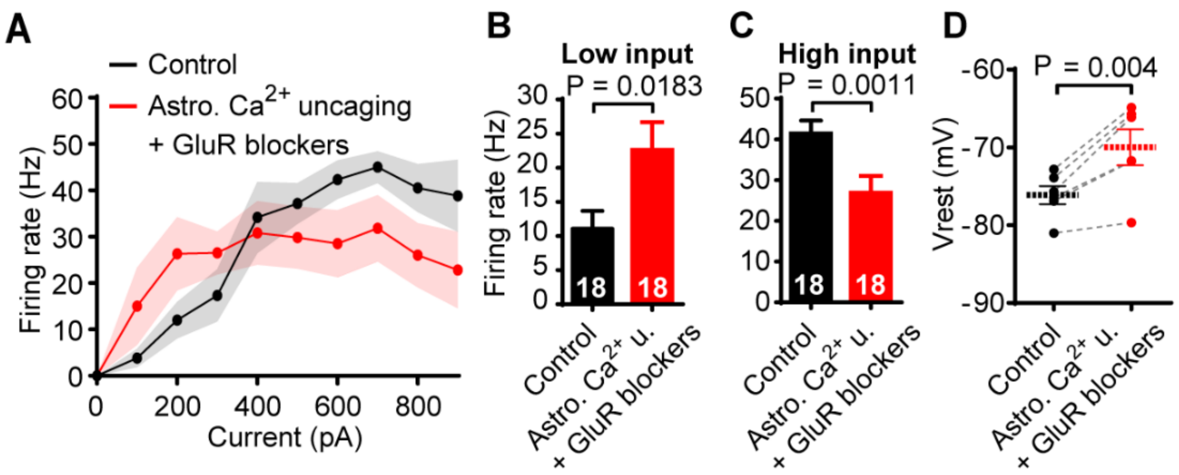

Fig. S5. Regulation of axonal excitability by astrocytes is not mediated by glutamate release. (**A**) Neuronal firing rate evoked by injecting 1 sec current steps in 100 pA increments before (black) and after astrocytic Ca^{2+} uncaging (red) in presence of 20 µM D-AP5 (NMDA-R antagonist), 10 µM DNQX (AMPA-R and KA-R antagonist), 50 µM MSPG (Group III and group II mGluR antagonist) and 1 µM NPS 2390 (Group I mGluR antagonist). (**B-C**) Firing rate change for (**B**) “low” (averaged over 100-300 pA, 18 current steps from 6 cells) or (**C**) “high” (700-900 pA, 18 current steps from 6 cells) input currents. (**D**) Mean resting potential before (black) and after astrocytic Ca^{2+} uncaging (red) in the presence of the iGluR and mGluR blockers in the bath. All data recapitulate the changes seen in the absence of the GluR blockers. All data are from rat.

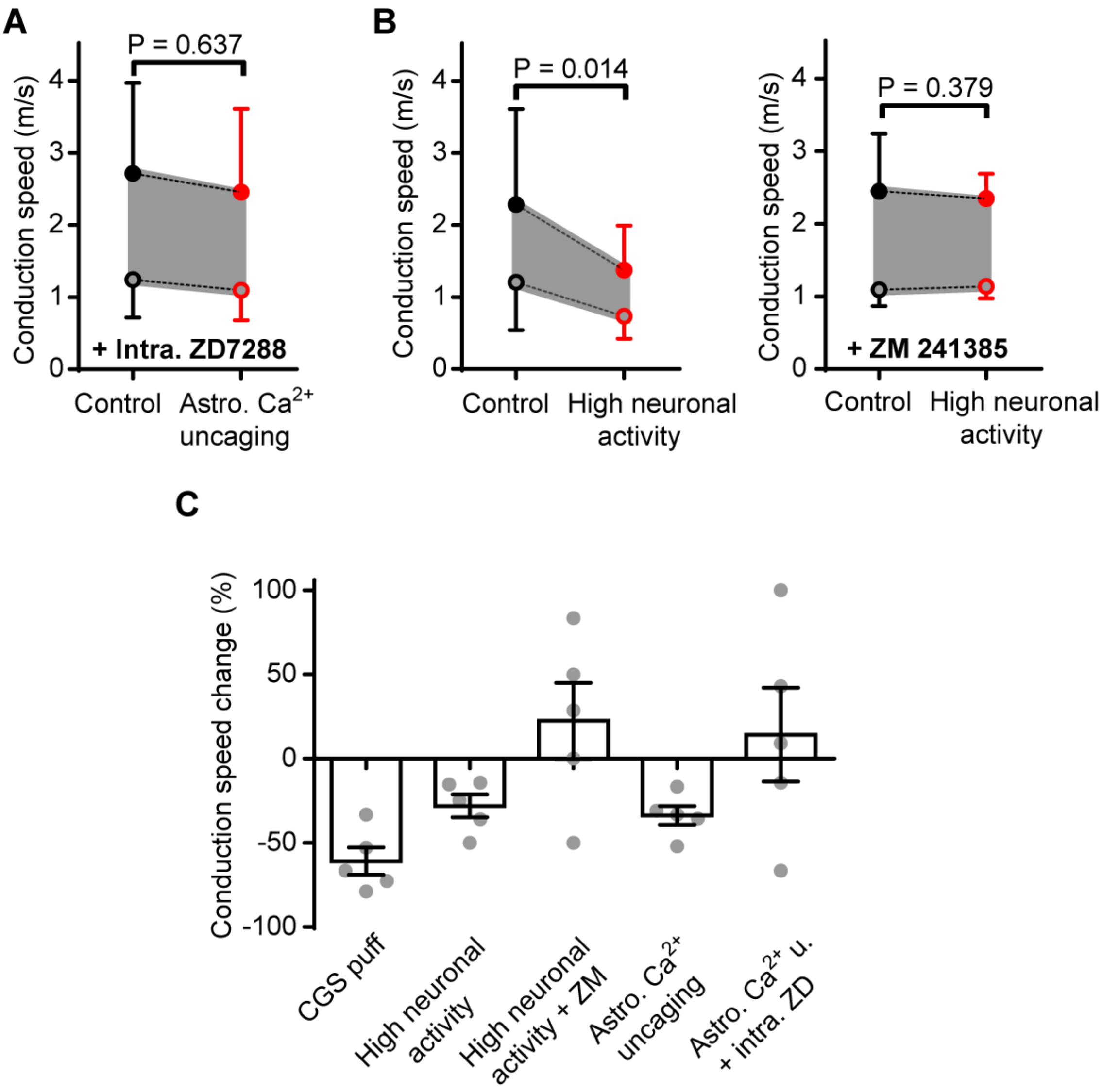

Fig. S6. Mechanisms underlying the modulation of axonal conduction speed. (**A**) Estimated axonal conduction speed (see text associated with Fig. 4E for assumptions made to obtain the plots with closed and open symbols) before and after astrocyte Ca^{2+} uncaging with ZD7288 (20 μM) dialysed into the neuron to block HCN channels (compare with Fig. 6I). In unmyelinated axons and some other neuronal types, axonal HCN channels can speed, rather than slow, the action potential (*13*, *14*). This presumably reflects HCN channels evoking either a smaller depolarization, or a depolarisation that overlaps differently with the activation and inactivation curves of the voltage-gated Na^+ channels. (**B**) Estimated axonal conduction speed before and after stimulating the neurons for 1 min at 30 Hz. Right panel shows conduction velocities with ZM 241385 (100 nM) bath applied to block A_{2a}Rs. (**C**) Summary of the experiments investigating the axonal conduction velocities. Data in panels A-B are from rat; panel C combines data from rats and mice (see legends in Fig. 4 and Fig. 6 for details).

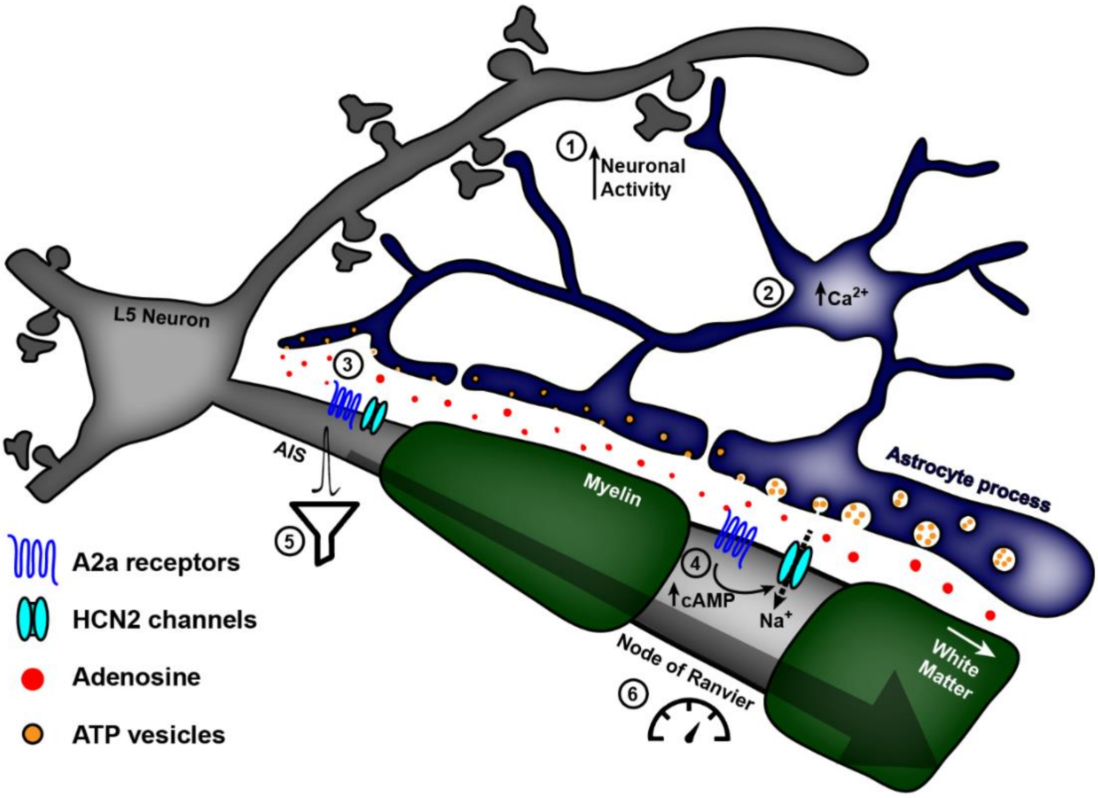

Fig. S7. Astrocytes modulate the properties of the AIS and of nodes of Ranvier in myelinated axons. Schematic diagram summarising how astrocyte-neuron interactions regulate axonal excitability and conduction speed. Neuronal activity can induce a $[Ca^{2+}]_i$ rise in astrocytes, preferentially in processes contacting dendrites (1). Strong triggers of astrocytic $[Ca^{2+}]_i$ rises, for example robust neuronal activity or uncaging, can lead to Ca^{2+} transients propagating to all astrocyte processes, including those contacting myelinated axons (2) at the AIS and at nodes of Ranvier. Astrocytic Ca^{2+} activity triggers the release from processes of vesicular ATP, which is converted into adenosine by extracellular ecto-ATPases (3). Adenosine activates A_{2a}Rs expressed highly in the AIS and nodes of Ranvier. This increases intracellular cAMP levels, thereby opening HCN2 channels (4). The resulting depolarization leads to two distinct physiological outcomes: (i) in the AIS, this mechanism filters the transmitted output (denoted by filter icon) by enhancing the action potential response to weak inputs but decreasing it to strong inputs (5); (ii) by acting on the nodes of Ranvier (6), it reduces the axonal conduction speed (denoted by speedometer). Schematic amended from ref. (*55*).

Supplementary movie captions

Movie S1. Neuronal activity triggers Ca^{2+} transients in astrocyte processes near axons and dendrites. An astrocyte was loaded with Fluo-4 to monitor its Ca^{2+} activity following neuron stimulation. The astrocyte processes contact the axon (left) and a dendrite (right) of a patch-clamped neuron dye-filled with Alexa 594 (same astrocyte as in Fig. 1D). In this experiment, robust firing of the neuron triggered Ca^{2+} transients in processes contacting the dendrite and the axon. Fluorescence at baseline was subtracted from all the time-lapse images.

Movie S2. Ca^{2+} uncaging in a periaxonal astrocyte. A L5 pyramidal neuron was whole cell patch-clamped and dye-filled with Alexa 594 (loaded in the left pipette, red), and an astrocyte (green) was loaded with NP-EGTA and Fluo-4 to uncage Ca^{2+} and monitor its level. Ca^{2+} was uncaged with 2-photon excitation at 720 nm targeted at the astrocyte soma. This led to a $[Ca^{2+}]_i$ rise in astrocyte processes, including those contacting the axon. Time-lapse images of the astrocyte were superimposed on a still image of the neuron to highlight the position of the astrocyte and its processes with regard to the axon. In this experiment, neuronal activity was monitored before and after astrocyte Ca^{2+} uncaging.

Movie S3. Astrocyte Ca^{2+} propagates in processes near the axon. Time-lapse images of the astrocyte shown in Fig. 6A. Ca^{2+} was uncaged at the soma by 2-photon excitation and is seen propagating in the processes going downwards, following the same direction of the axon of the L5 pyramidal neuron, towards the callosal white matter.
